# Commercial Crop Yields Reveal Strengths and Weaknesses for Organic Agriculture in the United States

**DOI:** 10.1371/journal.pone.0161673

**Published:** 2016-08-23

**Authors:** Andrew R. Kniss, Steven D. Savage, Randa Jabbour

**Affiliations:** 1 University of Wyoming, Department of Plant Sciences, Laramie, Wyoming, United States of America; 2 Independent consultant, Encinitas, California, United States of America; California State University Fresno, UNITED STATES

## Abstract

Land area devoted to organic agriculture has increased steadily over the last 20 years in the United States, and elsewhere around the world. A primary criticism of organic agriculture is lower yield compared to non-organic systems. Previous analyses documenting the yield deficiency in organic production have relied mostly on data generated under experimental conditions, but these studies do not necessarily reflect the full range of innovation or practical limitations that are part of commercial agriculture. The analysis we present here offers a new perspective, based on organic yield data collected from over 10,000 organic farmers representing nearly 800,000 hectares of organic farmland. We used publicly available data from the United States Department of Agriculture to estimate yield differences between organic and conventional production methods for the 2014 production year. Similar to previous work, organic crop yields in our analysis were lower than conventional crop yields for most crops. Averaged across all crops, organic yield averaged 80% of conventional yield. However, several crops had no significant difference in yields between organic and conventional production, and organic yields surpassed conventional yields for some hay crops. The organic to conventional yield ratio varied widely among crops, and in some cases, among locations within a crop. For soybean (*Glycine max*) and potato (*Solanum tuberosum*), organic yield was more similar to conventional yield in states where conventional yield was greatest. The opposite trend was observed for barley (*Hordeum vulgare*), wheat (*Triticum aestevum*), and hay crops, however, suggesting the geographical yield potential has an inconsistent effect on the organic yield gap.

## Introduction

Certified organic agricultural production area in the United States has increased steadily since the inception of the 1990 Organic Foods Production Act. Advantages of organic agriculture include economic benefits for producers [1] and increased provision of ecosystem services such as biological pest control and biodiversity conservation [2–4]. Sociocultural benefits such as quality of life for farming communities have been theorized, but research in this area of organic agriculture is limited [5].

One of the main criticisms of organic agriculture has consistently been lower crop yield compared to non-organic systems. Meta-analyses comparing yields of organic and conventionally grown crops have repeatedly demonstrated a yield gap between the two systems. Recently published meta-analyses report mean estimates across all crops varying from 19% to 25% lower yields in organic systems [6–8]. Critics of organic agriculture argue that society cannot justify being less efficient with arable land in the face of a rapidly growing human population. With respect to conservation interests, if more-efficient conventional farmers can match organic yields with 70% of the land, remaining land could be set aside for conservation and other environmental benefits [9–12]. However, yield gains have not been clearly linked with increased land set aside for conservation at the global or regional scale, thus the yield/conservation tradeoff is likely a false dichotomy not representative of the socioecological complexity of agricultural systems, with management decisions tied to markets and policy [13].

Yield differences between organic and conventional production vary with crop type and management practices. In their analysis of organic studies conducted world-wide, Seufert et al. [8] reported smaller yield gaps for organic fruit (3% lower than conventional) and oilseed crops (11% lower than conventional) and large gaps for organic cereals and vegetables (26% and 33% respectively). When studies were partitioned by plant type, organic legumes and perennials had more competitive yields than non-legumes and annuals, likely a result of more efficient nitrogen use by plants [8].

Meta-analyses of the published literature do not necessarily reflect the full range of innovation or practical limitations that are part of real-world commercial agriculture. Agricultural research, by necessity, often takes a reductionist approach in order to best isolate and quantify the effect of interest [14]. Additionally, equipment, labor availability, and scale of production is typically much different between research and commercial production. Although these differences may not necessarily bias yield differences between systems in any systematic way, there is always value in comparing estimates from controlled research with commercial production data. The analysis we present offers a new perspective, based on organic and conventional yield data reported to the United States Department of Agriculture (USDA) as part of their 2014 organic and agricultural producer surveys. The USDA data is a window into the range of farming operations and the best available measure of how the different production systems perform in a practical sense. USDA has made area and yield of organic and conventional crops, summarized at the state level, available to the public. Although this data set provides only a snapshot of agricultural production in the United States from one growing season, it represents actual commercial production rather than estimates from research studies. Data from field research stations and commercial farms are complementary, each with their own strengths and weaknesses [14]. The USDA survey data provides an opportunity to compare the findings of factorial research experiments with reported production yields. This rich data set offers yield comparisons from a diversity of crops and states, representing the breadth of organic and conventional agricultural production in the United States.

## Methods

We used state-level crop yield data from 2014 USDA surveys to estimate yield differences between organic and conventional production methods. The 2014 USDA Organic Survey had a target population of 16,992 organic farms in the United States, and achieved a response rate of 63% [15] using mail survey plus computer and phone follow-up interviews. Summarized survey data is publicly available [15]. Conventional yield data was obtained from the 2014 USDA-NASS December Agricultural Survey with a target population of over 83,000 farm operators, which is publicly available using Quick Stats [16]. From these two data sources, we assembled data pairs; each data pair consisted of the organic yield and conventional yield estimate for one crop from one state. This approach was used to control for different yield potential from different geographic regions. We acknowledge this approach may not be perfect, as organic area and conventional area are not necessarily in similar regions within a state. However, we assumed that the differences in yield potential due to geography within a state would be randomly distributed among states and crops, and thus, would not systematically bias our results in favor of one production system or the other.

Another shortcoming of our approach is that the breadth of organic production environments is not fully represented for some crops. For various reasons, USDA-NASS does not publish conventional production data for all crops in all states. Although conventional yield data were available for nearly all field and forage crops included in the organic survey, we could only assemble a limited number of data pairs for some fruit and vegetable crops. For example, organic spinach (*Spinacia oleracea*) yield data was available from 37 different states in 2014 (S1 Fig), but conventional spinach production was only reported for three of those states, resulting in only three data pairs. This lack of conventional yield data had the potential to bias our analysis. For nearly every crop where data pairs could not be assembled, average organic yield for states without conventional yield data was less than average organic yield where data pairs were available (S1 and S2 Figs). Therefore, our analysis was more likely to include states reporting above average organic crop yield. This bias is at least partially balanced by the fact that states with high production area (and, presumably, higher yield) for a particular crop are typically those included in USDA-NASS surveys for those crops. To reduce the potential for bias in our results, we excluded all crops with less than seven data pairs from our statistical analysis.

Yield data from the 2014 USDA-NASS survey was used as a proxy for conventional yield, even though it is possible that yield data from organic fields were included in the total yield estimates for some crops in some states. If organic acres are included in total yield estimates, our approach would slightly reduce the difference between the two systems. For this reason, our yield ratio estimates should be considered slightly conservative, since our results would be biased in favor of *less* difference between production systems. However, the difference between total yield reported in the USDA survey data and actual conventional yield will be negligible unless organic area is a large percentage of the total. Of the 519 data pairs in our data set, 477 had organic acres less than 10% of the total acres reported by USDA (S3 Fig).

Only four data pairs had organic area over 50% of the total area in the survey (Table 1). Those 4 observations included dry edible bean (*Phaseolus vulgaris*) in Arizona, squash (*Cucurbita spp*) and sweet corn (*Zea mays*) in Oregon, and spring wheat (*Triticum aestevum*) in Colorado. Organic squash area actually exceeded the total squash area reported from Oregon in 2014, and sweet corn was nearly the same.

**Table 1:** Comparisons where organic area was greater than 50% of total area reported.

Crop	Location	Organic hectares	Total hectares	Organic (% of total)
Dry edible bean	Arizona	2967	4413	67.2
Wheat (spring)	Colorado	1763	2834	62.2
Maize (sweet)	Oregon	1832	1984	92.3
Squash	Oregon	841	607	138.5

For 40 out of the 65 crops in our full data set, six or fewer states reported both conventional and organic yield data, so reliable confidence intervals of the yield ratio could not be calculated. Yield ratios for all crops, even those with fewer than seven data pairs, are provided in S4 through S7 Figs. We statistically analyzed yield data for 25 different crops that had at least seven data pairs.

One data pair was removed from the analysis. Apple (*Malus domestica*) production in Vermont reported by the USDA Organic Survey indicated an average yield of over 342,000 kg/ha, which was nearly an order of magnitude greater than any other apple yield observed for either conventional or organic production. We concluded this must have been a miscalculation.

The number of organic farms included in each data pair was used as a weighting factor in the analysis. In this way, yield estimates were given more weight in the analysis if they represented more farmers, since we had more confidence that those estimates were an accurate reflection of overall organic yield in that state. Data pairs were given less weight where the number of organic farmers contributing to the yield estimate was smaller. After removal of crops with less than seven data pairs, our analysis included yield estimates from 773,000 organic hectares (1.91 million organic acres) from 2014. This represents a much larger land area in our analysis compared to previous meta-analyses of published literature.

For each crop, we calculated the natural logarithm of the crop yield ratio (organic/conventional) from each state (data pair), weighted by the number of organic farms reporting from that state. The natural logarithm of the ratio was used to standardize the ratio to the same scale, regardless of whether conventional or organic production systems yielded more. Raw ratios would result in values always between 0 and 1 if organic yield was less than conventional, but values could range from 1 to infinity when organic yielded more. Taking the natural logarithm of the ratio re-scales the values around 0, and equalizes the magnitude of the distance from 0. For each crop, 95% confidence intervals around the natural logarithm of the yield ratio were calculated. Weighted means and confidence intervals were calculated by fitting a weighted least squares intercept-only linear model to the natural logarithm of the yield ratios for each crop. This was done using the *lm()*function in the statistical language R [17].

To simplify comparing our results with previously published meta-analyses [7, 8], we are presenting the data as organic to conventional crop yield ratios (i.e. back-transformed from log response ratios). Ratios less than 1.0 indicate organic crop yield was less than conventional crop yield, whereas ratios greater than 1.0 indicate organic crop yield was greater than conventional crop yield; organic and conventional crop yields were considered significantly different if the 95% confidence bars do not include 1.0. Median crop yield ratios (the ratio at which 50% of data pairs were greater and 50% were less than) have also been provided. In some cases, the mean and median crop yield ratio differed considerably within a crop. We have discussed these results in more detail in S1 Supplementary Information.

## Results and Discussion

Organic yields were lower than conventional yields for most crops. However, several crops had no significant difference in yields between organic and conventional production, and in a few examples, organic yields surpassed conventional yields. Across all crops and all states, organic yield averaged 80% of conventional yield. However, the yield ratio varied widely among crops, and in some cases, among states within a crop. Without more detail about the farms reporting yield data, it is impossible to conclude definitively the cause of the organic yield gap in any particular crop. The biggest production challenges organic farmers face relative to conventional farmers are with respect to fertility (especially nitrogen) due to a lack of synthetic fertilizers, and pest management (weeds, insects, and pathogens) due to a lack of synthetic pesticides [6]. These production challenges are likely responsible for the organic yield gap in most of the crops we analyzed, though the relative contribution of each may differ. Because yield data is reported and analyzed at the state level, any discussion on the specific cause of yield differences between organic and conventional production of a particular crop would be speculation. For this reason, we have refrained from delving too deeply into any specific crop in our analysis, and instead focus on broader trends, though some more detailed discussion and data can be found in S1 Supplementary Information.

**Fig 1:**
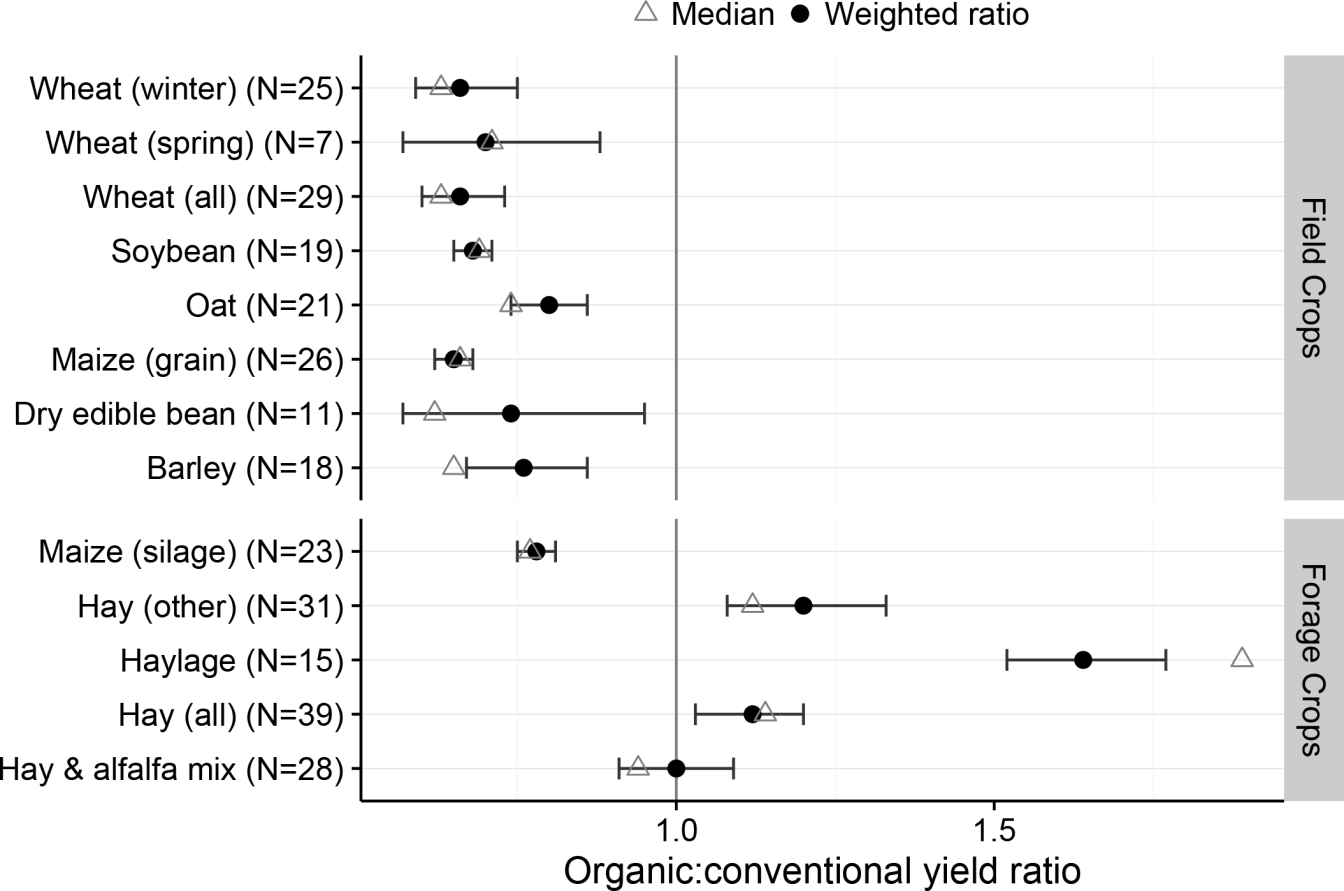
Field and forage crop yield ratio of organic to conventional yield from states reporting both organic and conventional yield data in 2014 USDA surveys. Circles represent weighted ratio mean estimates, error bars represent 95% confidence limits for the weighted ratio; triangles represent the median crop yield ratio for all states included in the analysis.

Organic crop yields were significantly less than conventional yields for 9 of 13 field and forage crops (Fig 1). Organic wheat yield was significantly less than conventional wheat for both spring and winter types. Combined over types, organic wheat yielded 66% of conventional yield. Organic soybean yielded 68% of conventional. The organic cereal crops maize and barley yielded 65% and 76% of conventional yield, respectively. The organic oat (*Avena sativa*) yield gap was less, but organic still only produced 80% of conventional oat yield.

Lower organic crop yields in the field crops in our analysis are likely associated with the challenges of balancing soil quality and weed management in organic grain production [18, 19]. Organic farmers have long reported major challenges with weed management [20], with recent reports specifying problematic perennials such as field bindweed (*Convolvulus arvensis*) and Canada thistle (*Cirsium arvense*) [21]. Organic agriculture has been criticized for use of tillage and associated negative environmental impacts such as soil erosion [12]. Of the organic farmers surveyed in the 2014 Census (and whose yield data are included here), 40% reported use of no-till or minimum till practices [15]. Reduced tillage in organic grain systems often results in improved soil quality [22] but with the trade-off of more perennial weeds [23] or inadequate nitrogen for non-legume grain crops [24]. In regional surveys of organic farmers (of all types, not just grain producers), both annual and perennial weeds continue to be mentioned as most problematic [25–27] although organic farmer knowledge has been associated with lower proportions of problematic annuals [27]. It is unclear why such a difference between states was observed with organic to conventional yield ratio in dry edible bean and soybean. Since they are legumes, nitrogen deficiency should play a minimal role in contrast to many other organic crops, as long as the seed is inoculated with the appropriate rhizobium species. For dry bean production, Idaho and Colorado represent relatively similar growing environments with respect to dry edible bean production, and conventional yields were similar between these two states (S1 Supplementary Information). Even though conventional yields were similar, organic to conventional yield ratios of 1.11 and 0.45, were observed in Idaho and Colorado, respectively, because organic dry bean yield was much lower in Colorado.

As a group, organic hay crops yielded similarly or significantly greater than conventional hay crops (Fig 1), though this was not true for the annual crop maize harvested for silage. Seufert et al. [8] suggested in their meta-analysis that perennial crops and legumes tended to produce organic crop yields more similar to conventional crop yields compared to other organic crops, which is supported by the superior performance of the organic perennial hay crops compared to the annual silage crop in our analysis. Most crops grown for hay are perennial, and alfalfa (*Medicago sativa*) is both a perennial and a legume. These traits should give organic hay a relative advantage compared to many other organic crops.

In 2010, the National Organic Program specified new regulations about ruminant production, stating that at least 30% of dry matter intake must be provided from grazing pasture or from “residual forage” cut and laying in pasture during the grazing season [28]. Thus, there is high demand and motivation to provide high-quality organic forage for organic dairy and meat production which may drive producers to increase management intensity in these systems. Hay and forage crops also present an opportunity to incorporate species diversity into the cropping system with relative ease through species mixtures. Increased species diversity has been linked to greater fodder productivity [29], and supporting biodiversity is encouraged by the National Organic Program [30–32].

**Fig 2:**
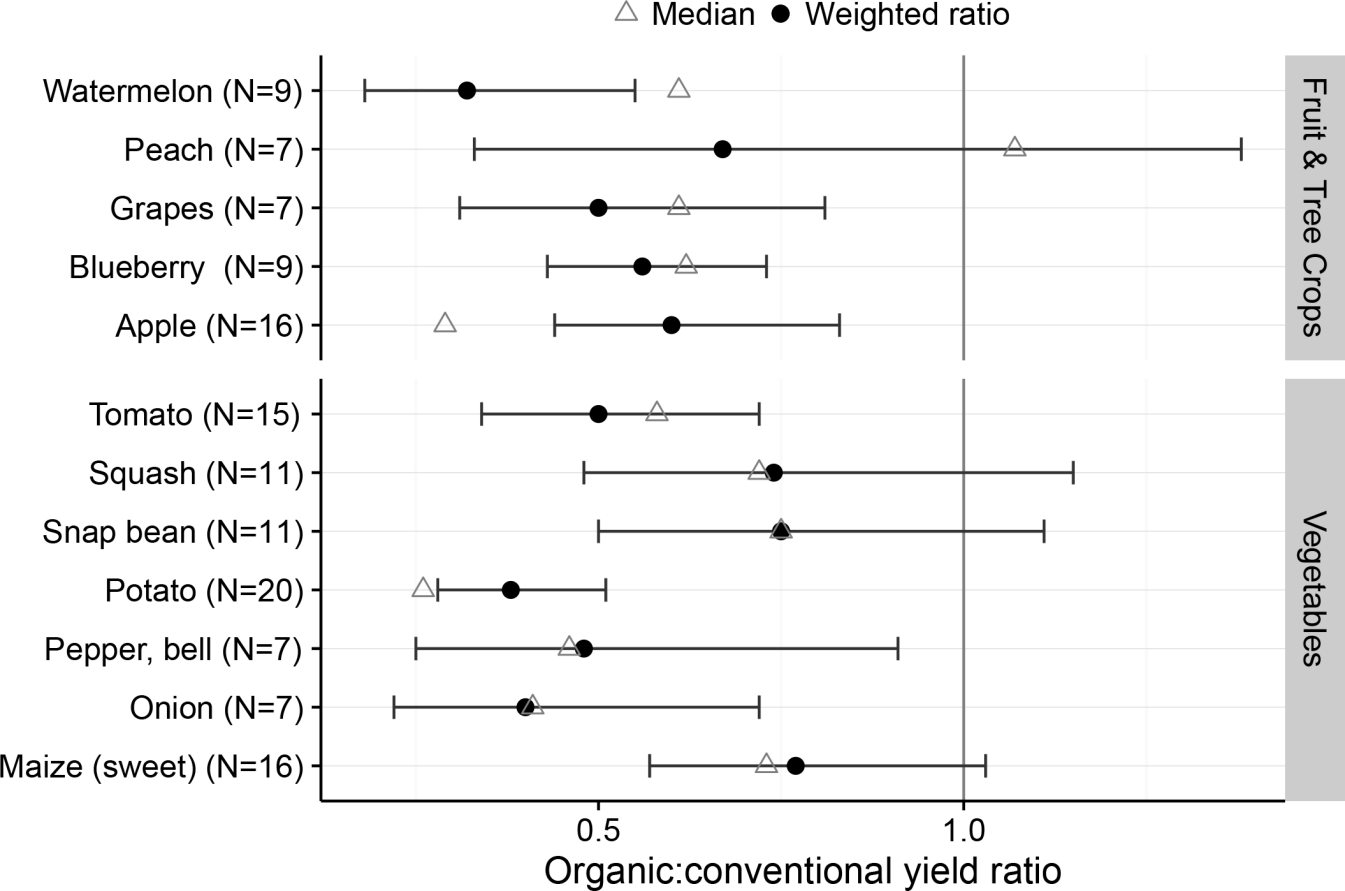
Fruit and vegetable yield ratio of organic to total yield from states reporting organic yields in the 2014 USDA survey. Circles represent weighted ratio mean estimates, error bars represent 95% confidence limits for the weighted ratio; triangles represent the median crop yield ratio for all states included in the analysis.

Previous work [7, 8] has suggested that organic vegetables tend to perform worse relative to conventional practices compared to other crop types. In our analysis, organic vegetable crop yields ranged from 38% (potato) to 77% (sweet maize) of conventional yields (Fig 2). Organic squash, snap bean (*Phaseolus vulgaris*), sweet maize, and peach (*Prunus persica*) yields were not statistically different from conventional, while average yield of all other organic vegetables (tomato (*Solanum lycopersicum*), potato, bell pepper (*Capsicum anuum*), and onion (*Allium cepa*)) and fruits (watermelon (*Citrullus lanatus*), grape (*Vitis vinifera*), blueberry (*Vaccinium myrtillus*), and apple) were less than conventional.

Organic fruit and vegetable production is often associated with direct marketing to consumers through either farmers markets or community-supported agriculture (CSA) operations. Of the respondents to the 2014 organic survey, 6,382 (37.6%) reported marketing to consumers directly, in contrast to 6.9% of all United States farms reporting direct-to-consumer sales [33, 34]. Pest management, especially insect and fungal pathogens, can be particularly problematic for organic producers selling into fresh markets, as there are far fewer approved pesticides available for use in organic agriculture. Insect and disease damaged fruits and vegetables can quickly become unmarketable, and this might explain the relatively low organic yields of fruit and vegetable crops compared to their conventional counterparts.

### Comparison with previous analyses

**Fig 3:**
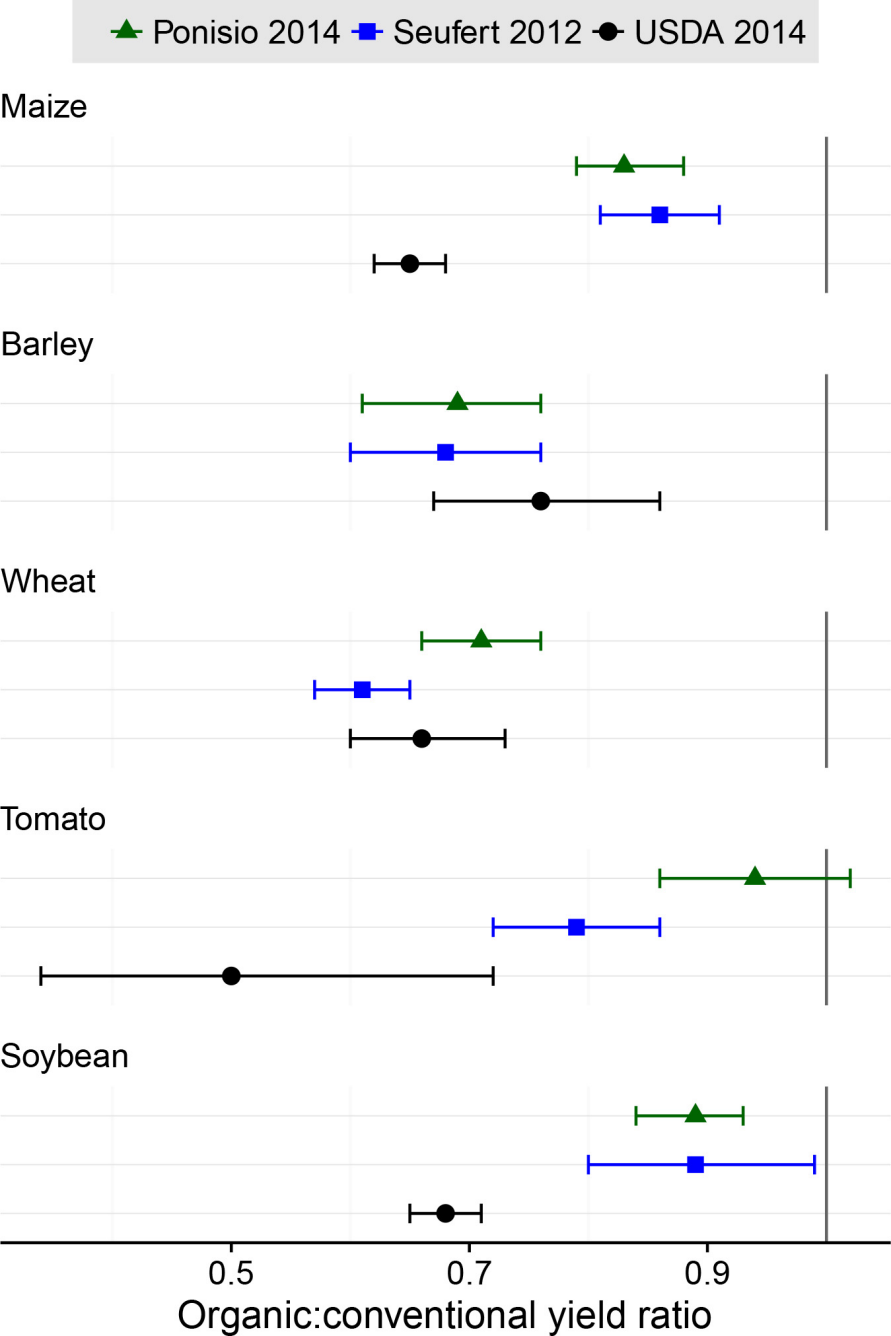
Relative yield of organic maize, barley, wheat, tomato, and soybean. Green triangles adapted from meta-analysis results presented by Ponisio et al. (2015); blue squares adapted from meta-analysis results presented by Seufert et al. (2012); black circles represent our analysis of USDA data from 2014. Points are the ratio of organic:conventional yields, bars represent 95% confidence intervals around those estimates.

As part of a large meta-analysis of organic yield studies, Seufert et al. [8] presented wheat, tomato, soybean, maize, and barley yield ratios. Ponisio et al. [7] then re-analyzed much of the same data used by Seufert et al. We have re-created their previous yield ratio estimates and 95% confidence intervals here for direct comparison with our estimates based on 2014 USDA yield data (Fig 3). The Seufert et al. and Ponisio et al. analyses used comparisons from previously published experiments and surveys, and therefore, may not represent actual practice as well as the USDA survey data in our analysis. In addition, Seufert et al. and Ponisio et al. included research from around the world, including developing countries, while USDA estimates are exclusive to the United States.

The main limitations of the USDA data are the potential for responder bias and the absence of relevant information that could help explain yield variation. We do not know which producers responded to the survey due to confidentiality, nor how representative they are of the producers in their state. We cannot determine whether the 63% of organic producers who responded to the survey are more or less productive, growing low or high diversity of crops, or on different soil types. Experimental yield comparisons, such as those included in Seufert [8] and Ponisio [7], are better able to control for sources of variation such as soil type, climate, and surrounding landscape. Because the data used by Seufert et al. and Ponisio et al. are independent of our own data, comparing yield gaps from yields reported by United States producers to those presented through previous meta-analyses allows us to evaluate the generality of our findings.

Organic crop yield for all five crops in Fig 3 were significantly less than conventional crop yield in our analysis based on USDA estimates, which is similar to results presented by Seufert et al. [8], and with the exception of tomato, also similar to Ponisio’s [7] meta-analysis. For maize, soybean, and tomato, our analysis of UDSA data shows an organic yield gap that is substantially greater than previous estimates; that is, commercial organic yields for these crops are further behind conventional yields than previous analyses suggest. There are, our analysis indicates, still improvements to be made in commercial organic production of maize, tomato, and soybean for these crops to meet the results obtained mostly under experimental conditions. For wheat and barley, USDA yield estimates from 2014 suggest yield ratios similar to the estimates from Seufert et al. [8] and Ponisio [7].

Although our data agree with previous work showing lower yields in organic production systems in general, our data suggest that commercial hay crops produced significantly greater yield when produced in an organically managed system. This is contrary to Seufert et al. [8] and Ponisio et al. [7] who did not find evidence for greater yield under organic management. Seufert suggested that the organic yield gap was less for legume and perennial crops compared to non-legume and annual crops, respectively. In contrast, Ponisio et al. concluded there were not major differences between annual vs perennial crops, nor with legume vs non-legume crops with respect to the organic yield gap. Our analysis agrees more closely with Seufert et al., showing that annuals and non-legumes fared worse under organic management compared to perennials and legumes, since hay crops tend to be primarily perennial and also include legumes (Fig 1).

It is important to note, however, that broad categories (like annual vs perennial) will be greatly influenced by which crops are included in the analysis. These comparisons are, therefore, fairly dubious. For example, grapes and haylage are both perennial crops, but the organic yield ratios for these crops varied dramatically (50% and 164% of conventional yields, respectively). So to generalize that perennials fare better than annuals under organic management would be misleading without greater context. Our analysis of USDA data provides estimates for annual, perennial, and non-legume crops that are quite different from Ponisio et al. [7], but this difference may be largely due to the crops that were included in each analysis (S8 Fig).

Previous work by dePonti et al. [6] hypothesized that the difference between organic and conventional yields would increase as conventional yield for the crop increased. Their hypothesis stemmed from the idea that organic systems are more limited by fertility and pest management options relative to conventional systems; so as conventional yields approach their water-limited yield potential, organic systems would lag further behind. They found weak evidence to support their hypothesis, as the organic to conventional yield ratio decreased as conventional yield increased, though the relationship was only statistically significant for two crops (soybean and wheat).

**Fig 4:**
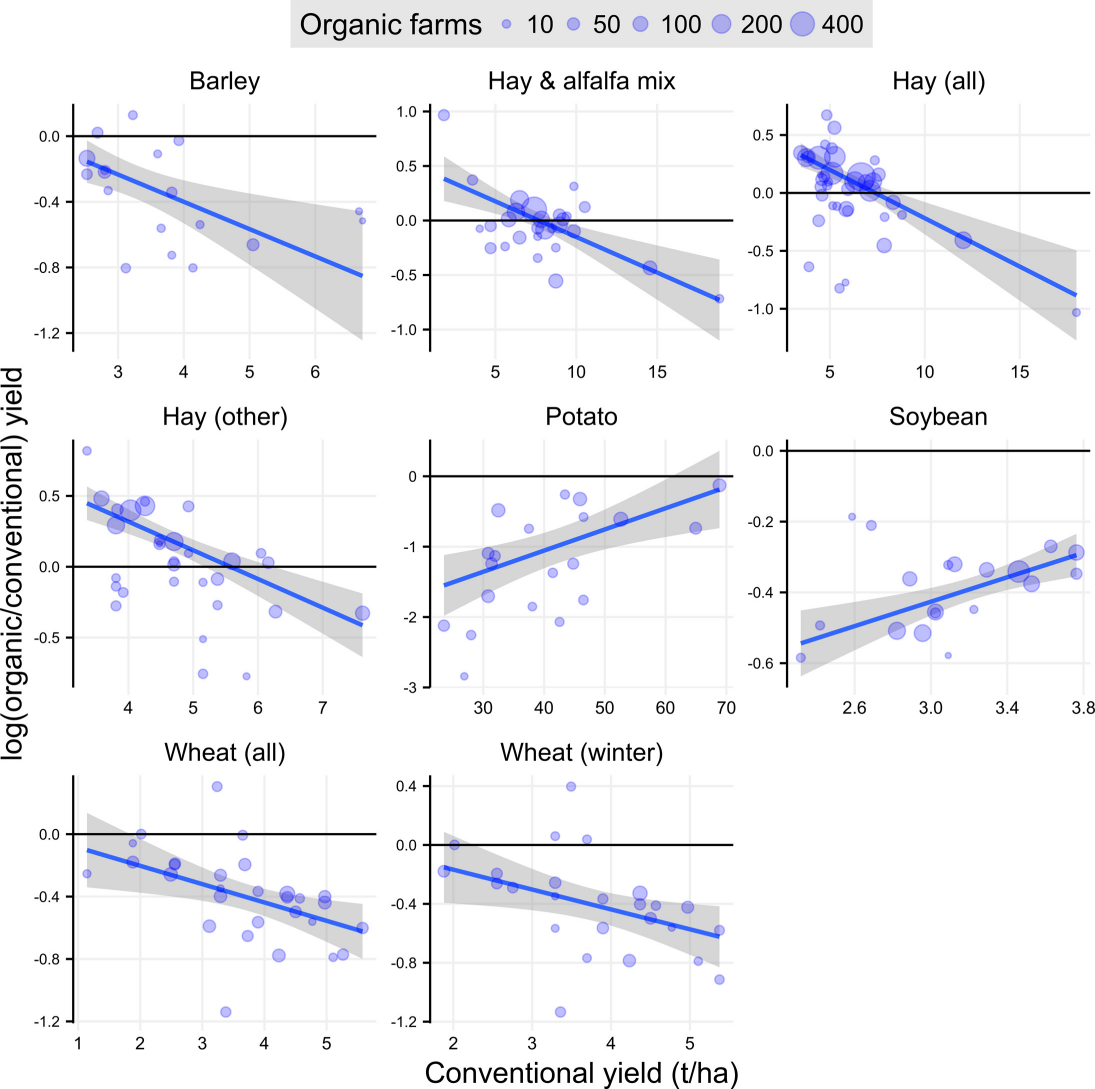
Relationship between organic to conventional crop yield ratio and conventional crop yield for eight crops. Circles each represent one state reporting both organic and conventional crop yield data to the USDA in 2014; size of the circles is proportional to the number of organic farmers reporting crop yield data from that state. Black horizontal line at zero represents no yield difference between organic and conventional crop yield. Blue line is the weighted least squares regression line, using the number of organic farms reporting in each state as the weighting factor; gray shaded area is the 95% confidence interval around the weighted regression line. Slope estimates, p-values, and R^2^ values can be found in Table 2.

We conducted a similar analysis to de Ponti et al. [6] for the 25 crops with at least seven data pairs, using a weighted regression to determine whether the organic to conventional yield ratio was related to conventional yield. Out of the 25 crops we analyzed, eight showed a significant relationship between organic to conventional crop yield ratio and conventional crop yield, including soybean and wheat, the two crops that were significant in the de Ponti analysis (Fig 4). Of those eight crops, six showed a decreasing trend, similar to that observed by de Ponti et al. However, contrary to de Ponti’s hypothesis, soybean and potato showed an increasing trend in our analysis, suggesting that in locations with greater conventional yields, the organic yield gap was lowest. If the statistical significance is ignored and only the direction of the slope (increasing or decreasing) is considered, 15 out of 25 crops had negative slopes compared to 10 with positive slopes (Table 2). The relationship between the organic yield gap and conventional yield potential does not appear to generalize well across different crops, and in fact, can be completely different depending on the crop of interest.

**Table 2:** Weighted least squares regression slope, standard error (S.E.), p-value, and R^2^ for 25 crops investigating the relationship between ln(organic:conventional crop yield) as the dependent variable and conventional crop yield (ton/ha) as the independent variable using 2014 USDA survey data.

Crop	Slope	S.E.	P-value	R^2^
Apple	0.007	0.009	0.468	0.038
Barley	-0.166	0.052	0.005	0.393
Blueberry	-0.031	0.033	0.373	0.114
Dry edible bean	-0.508	0.396	0.231	0.155
Grapes	0.001	0.061	0.982	0.000
Hay & alfalfa mix	-0.065	0.016	0.000	0.393
Hay (all)	-0.083	0.016	0.000	0.425
Haylage	0.002	0.023	0.921	0.001
Hay (other)	-0.203	0.035	0.000	0.530
Maize (grain)	-0.038	0.025	0.136	0.090
Maize (silage)	0.004	0.004	0.393	0.035
Maize (sweet)	0.004	0.033	0.894	0.001
Oat	-0.028	0.117	0.816	0.003
Onion	0.020	0.017	0.309	0.204
Peach	-0.029	0.028	0.351	0.175
Pepper, bell	0.025	0.022	0.310	0.203
Potato	0.030	0.009	0.003	0.389
Snap bean	0.023	0.060	0.709	0.016
Soybean	0.173	0.045	0.001	0.459
Squash	-0.054	0.033	0.132	0.234
Tomato	-0.010	0.011	0.402	0.055
Watermelon	-0.003	0.014	0.848	0.006
Wheat (all)	-0.117	0.041	0.009	0.229
Wheat (spring)	-0.165	0.143	0.300	0.211
Wheat (winter)	-0.135	0.054	0.021	0.212

A majority of organically-produced crops in our analysis produced significantly lower yield compared to conventional systems. But agricultural systems should not be judged on yield alone. A primary goal for agriculture of the future should be to produce enough food to feed a growing population, and to do so while minimizing the negative impacts of that production. Organic agriculture has demonstrable benefits to the environment on a per unit area basis, however, those benefits are often negated or reversed on a per unit production basis because organic systems tend to yield less per area [5].

In England, Hodgson et al. [35] estimated that organic yields must be at least 87% of conventional yields to make organic production better for butterfly abundance (a proxy for ecosystem health), as long as the land spared by conventional production was used for nature reserves. Detractors of organic production often cite “land sparing” as a primary benefit due to the improved yields observed in conventional agriculture. But land sparing (increasing production to set aside land for nature) only works if land is actually spared due to increased production. In the US, while yield of major staple crops like maize, wheat, and soybean have continued to increase using conventional production practices, land devoted to conservation reserves has decreased significantly since 2007 [36]. If large areas of land are not set aside, then a land sharing approach may be warranted instead. Hodgson et al. [35] estimated that without large conservation areas, optimal land use would favor organic as long as organic yields were at least 35% of conventional (land sharing). This is because organic production practices in some cropping systems tend to favor pollinators and other beneficial species compared to conventionally managed fields [35, 37]. Kremen [13] recently argued for a “both-and” framework, rather than choosing between land sparing and land sharing. She proposed that scientists focus research on evaluating whether specific management practices can increase biodiversity without compromising yield. This future research aim is applicable to both organic and conventional agriculture, as a spectrum of management practices exist in farms of each classification.

The reasons for food insecurity around the world are varied and complex, and go far beyond just yield. Even so, a dramatic, sustained reduction in crop yield could be devastating to food security, even in developed countries, making a rapid and complete switch to organic agriculture unwise. Unless other inefficiencies in our food systems are corrected (like food waste, food distribution, and meat-intensive diets), we are likely to need continued yield increases into the future to feed a growing population. Based on our estimates, if all US wheat production were grown organically, an additional 12.4 million hectares (30.6 million acres) would be needed to match 2014 production levels in the U.S., unless the organic yield gap can be narrowed. Current annual production of some crops (like wheat, corn, and soybean) are greater than annual domestic consumption in the U.S., allowing for export. Given world population projections and diet trends, maintaining current production levels in developed countries (while continuing to increase production in developing countries) will likely be the minimum required for a food-secure world.

There are a wide variety of behaviors and experience levels within both organic and conventional production. Where a farmer fits into that spectrum will drive their productivity and sustainability in economic, environmental, and social dimensions. Although the long-term sustainability of organic production is debated nearly as often as conventional practices, many consumers buy organic food because of the perceived environmental benefits [38]. Other sustainability marketing efforts that go beyond organic production have been proposed (like Whole Foods “Responsibly Grown” and the Field to Market “Fieldprint” programs). However, these programs have not gained wide acceptance or recognition.

Farmer adoption of organic agriculture is likely linked to geography. “Hotspot” areas of organic adoption have been documented in England, associated with physical characteristics such as soil type and altitude as well as socioeconomic characteristics like population size or distance from urban centers [39]. These hotspots were not associated with higher organic yields, but rather occurred in lower yielding regions for both conventional and organic production [39, 40]. Geographic clustering occurs in the United States as well. Of the organic farms surveyed in 2014, California, Wisconsin, New York, Washington, and Pennsylvania had the highest number of operators reporting, respectively. Operators from these five states represented 13,423 (45%) of the organic producers surveyed. States vary according to climate and growing conditions but may also vary according to available regional markets, outreach and education on the topic of organic agriculture, and farmer associations.

In addition to geographic drivers of variation between states, organic farm location within a state may also be geographically clustered, with clusters potentially in distinct landscapes or soil types that could alter productivity. For example, California was the largest grape producer for both organic and conventional in our analysis. Organic wine grapes are often produced in low yielding coastal areas, while conventional grapes are also grown in the higher yielding Central Valley of California. This could potentially bias our analysis in favor of conventional production in that instance. However, we were unable to access information about the specific locations within states of the respondents, a limitation of this data set, thus we cannot test for this potential source of bias explicitly. Prior research from England suggests there are complex drivers and impacts of spatial clustering of organic farms that may or may not relate to organic crop yield gaps [39, 40]. More research on the geography of organic agriculture in the United States is needed to determine whether clustering could drive the yield trends in our study.

USDA data from the 2014 surveys illustrates the breadth and diversity of organic production in the United States. To efficiently produce not just organic crops, but all crops, scientists, farmers, and Extension professionals would benefit from cross-regional comparisons and collaborations. Many unanswered questions remain regarding multifunctional agriculture of both organic and conventional systems, and future research should explore not only yield outcomes but also environmental impacts of management decisions [13]. In particular, most crops consistently illustrate large organic yield gaps and merit more organic-focused research to support these producers. In particular, efforts to improve available varieties for use in organic production may result in yield improvement via improved nutrient acquisition, pest resistance, competitive traits, or other gene by environment interactions [41]. Furthermore, examination of commonalities and differences between organic and conventional production practices in states with the best and worst yield ratios could be informative. Detailed knowledge of these specific production systems is necessary to investigate these comparisons, presenting an important opportunity for cross-commodity collaboration as well. Our findings support the importance of research funding at the federal level to facilitate such collaborations which may be otherwise difficult to execute but which are crucial to improving the sustainability of US agriculture.

## Supporting Information

S1 Data Organic and conventional yield data compiled from 2014 USDA surveys for analysis. (CSV)

S2 Data Yield data from 2014 USDA surveys compiled for analysis, including organic yield data without corresponding data pairs. This data was used to create S1 and S2 Figs.
(CSV)

S3 Data Data from Seufert et al (2012) and Ponisio et al (2014) for five crops used to create Fig 3. (CSV)

S4 Data Data from Seufert et al. (2012) and Ponisio et al. (2014) for different crop groups used to create S8 Fig. (CSV)

S1 Fig Organic vegetable yields from the 2014 USDA Organic Survey. **Triangles represent states reporting organic yield where no conventional yield data were available.** Circles represent states with both organic and conventional data available (data pair). Panel A: each point represents the crop mean from states with and without data pairs. Panel B: each point represents the organic yield reported from each individual state.
(EPS)

S2 Fig Organic fruit and tree crop yields from the 2014 USDA Organic Survey. **Triangles represent states reporting organic yield where no conventional yield data were available.** Circles represent states with both organic and conventional data available (data pair). Panel A: each point represents the crop mean from states with and without data pairs. Panel B: each point represents the organic yield reported from each individual state.
(EPS)

S3 Fig Distribution of organic acres as percentage of total acres for each crop. Histogram excludes four data pairs with organic acres over 60% of conventional acres, which are included in Table 1.
(EPS)

S4 Fig Distribution of the natural logarithm of the organic to conventional yield ratio for all field crops. (EPS)

S5 Fig Distribution of the natural logarithm of the organic to conventional yield ratio for all forage crops. (EPS)

S6 Fig Distribution of the natural logarithm of the organic to conventional yield ratio for all fruit and tree crops. (EPS)

S7 Fig Distribution of the natural logarithm of the organic to conventional yield ratio for all vegetable crops. (EPS)

S8 Fig Influence of nitrogen fixation potential and crop longevity on organic:conventional yield ratio. Green triangles adapted from Ponisio (2014); blue squares adapted from Seufert (2012); black circles represent analysis of USDA yield data (2014). Points are the ratio of organic:conventional yield, error bars represent 95% confidence intervals around those estimates.
(EPS)

S1 Supplementary Information Tabular estimates for figures, and summarized data for crops not included in the statistical analysis. (HTML)
