# Correction: Commercial Crop Yields Reveal Strengths and Weaknesses for Organic Agriculture in the United States

**DOI:** 10.1371/journal.pone.0165851

**Published:** 2016-11-08

**Authors:** Andrew R. Kniss, Steven D. Savage, Randa Jabbour

There is an error in the seventh sentence of the Abstract. The correct sentence is: Averaged across all crops, organic yield averaged 67% of conventional yield.

There is an error in the third sentence of the first paragraph of the Results and Discussion. The correct sentence is: Across all crops and all states, organic yield averaged 67% of conventional yield.

There is an error in the first sentence of the second paragraph of the Results and Discussion. The correct sentence is: Organic crop yields were significantly less than conventional yields for 10 of 13 field and forage crops (Fig 1).

**Fig 1:**
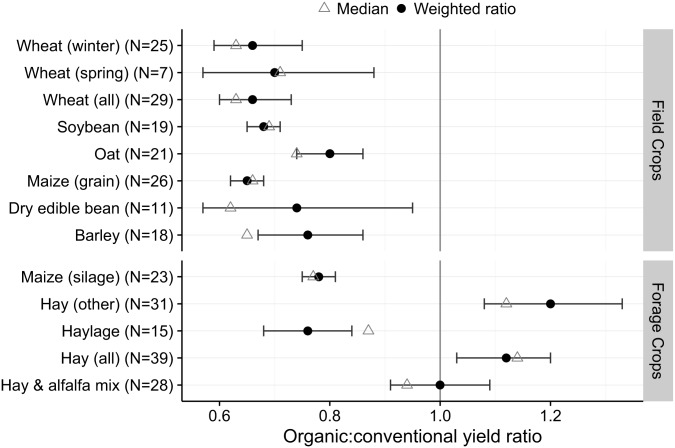
Field and forage crop yield ratio of organic to conventional yield from states reporting both organic and conventional yield data in 2014 USDA surveys.

There is an error in the first sentence of the fourth paragraph of the Results and Discussion. The correct sentence is: As a group, organic hay crops yielded similarly or significantly greater than conventional hay crops (Fig 1), though this was not true for the annual crop maize harvested for silage or haylage.

There are errors in the third sentence of the fifth paragraph of the Comparison with Previous Analyses section in the Results and Discussion. The correct sentence is: For example, grapes and alfalfa are both perennial crops, but the organic yield ratios for these crops varied dramatically (50% and 100% of conventional yields, respectively).

There are errors in the fourth sentence of the seventh paragraph of the Comparison with Previous Analyses section in the Results and Discussion. The correct sentence is: If the statistical significance is ignored and only the direction of the slope (increasing or decreasing) is considered, 16 out of 25 crops had negative slopes compared to 9 with positive slopes (Table 2).

Fig 1 is incorrect. The authors have provided a corrected version here.

Circles represent weighted ratio mean estimates, error bars represent 95% confidence limits for the weighted ratio; triangles represent the median crop yield ratio for all states included in the analysis.

There is an error in Table 2. The values listed in the row 9 "Haylage" are incorrect. Please see the corrected Table 2 here.

**Table 2:** Weighted least squares regression slope, standard error (S.E.), p-value, and R2 for 25 crops investigating the relationship between ln(organic:conventional crop yield) as the dependent variable and conventional crop yield (ton/ha) as the independent variable using 2014 USDA survey data.

Crop	Slope	S.E.	P-value	R^2^
Apple	0.007	0.009	0.468	0.038
Barley	-0.166	0.052	0.005	0.393
Blueberry	-0.031	0.033	0.373	0.114
Dry edible bean	-0.508	0.396	0.231	0.155
Grapes	0.001	0.061	0.982	0.000
Hay & alfalfa mix	-0.065	0.016	0.000	0.393
Hay (all)	-0.083	0.016	0.000	0.425
Haylage	-0.003	0.015	0.872	0.0002
Hay (other)	-0.203	0.035	0.000	0.530
Maize (grain)	-0.038	0.025	0.136	0.090
Maize (silage)	0.004	0.004	0.393	0.035
Maize (sweet)	0.004	0.033	0.894	0.001
Oat	-0.028	0.117	0.816	0.003
Onion	0.020	0.017	0.309	0.204
Peach	-0.029	0.028	0.351	0.175
Pepper, bell	0.025	0.022	0.310	0.203
Potato	0.030	0.009	0.003	0.389
Snap bean	0.023	0.060	0.709	0.016
Soybean	0.173	0.045	0.001	0.459
Squash	-0.054	0.033	0.132	0.234
Tomato	-0.010	0.011	0.402	0.055
Watermelon	-0.003	0.014	0.848	0.006
Wheat (all)	-0.117	0.041	0.009	0.229
Wheat (spring)	-0.165	0.143	0.300	0.211
Wheat (winter)	-0.135	0.054	0.021	0.212

S1 Data is incorrect. The authors have provided a corrected version here.

S5 Fig is incorrect. The authors have provided a corrected version here.

S8 Fig is incorrect. The authors have provided a corrected version here.

S1 Supplementary Information is incorrect. The authors have provided a corrected version here.

## Supporting Information

S1 Data Organic and conventional yield data compiled from 2014 USDA surveys for analysis. (XLSX)

S5 Fig Distribution of the natural logarithm of the organic to conventional yield ratio for all forage crops. (EPS)

S8 Fig Influence of nitrogen fixation potential and crop longevity on organic:conventional yield ratio. Green triangles adapted from Ponisio (2014); blue squares adapted from Seufert (2012); black circles represent analysis of USDA yield data (2014). Points are the ratio of organic:conventional yield, error bars represent 95% confidence intervals around those estimates.
(EPS)

S1 Supplementary Information Tabular estimates for figures, and summarized data for crops not included in the statistical analysis. (HTML)
