# Supplementary material for: Commercial Crop Yields Reveal Strengths and Weaknesses for Organic Agriculture in the United States
Source: PLoS One. 2016 Aug 23;11(8):e0161673. doi: 10.1371/journal.pone.0161673 (PMC4995028; doi:10.1371/journal.pone.0161673)
Supplement: S1 Supplementary Information — (HTML) [file pone.0161673.s013.html]

Commercial crop yields reveal strengths and weaknesses for organic agriculture in the United States - Supplementary material


# Commercial crop yields reveal strengths and weaknesses for organic agriculture in the United States - Supplementary material

#### *Andrew Kniss, Steve Savage, & Randa Jabbour*

# Mean organic crop yields (ton/ha) by conventional crop yield data pair availability (Figure S1 & S2)

| Group | Crop | DataPair | YieldMean |
| --- | --- | --- | --- |
| Field Crops | Barley | Conventional data pair available | 2.64 |
| Field Crops | Barley | No conventional data pair | 1.97 |
| Field Crops | Dry edible bean | Conventional data pair available | 1.57 |
| Field Crops | Dry edible bean | No conventional data pair | 1.05 |
| Field Crops | Maize (grain) | Conventional data pair available | 7.08 |
| Field Crops | Maize (grain) | No conventional data pair | 6.44 |
| Field Crops | Maize (silage) | Conventional data pair available | 36.35 |
| Field Crops | Oat | Conventional data pair available | 2.01 |
| Field Crops | Oat | No conventional data pair | 2.03 |
| Field Crops | Sorghum (silage) | Conventional data pair available | 26.61 |
| Field Crops | Sorghum (silage) | No conventional data pair | 24.60 |
| Field Crops | Soybean | Conventional data pair available | 2.12 |
| Field Crops | Soybean | No conventional data pair | 2.03 |
| Field Crops | Wheat (all) | Conventional data pair available | 2.44 |
| Field Crops | Wheat (all) | No conventional data pair | 1.88 |
| Fruit & Tree Crops | Apple | Conventional data pair available | 12.19 |
| Fruit & Tree Crops | Apple | No conventional data pair | 10.26 |
| Fruit & Tree Crops | Avocado | Conventional data pair available | 5.70 |
| Fruit & Tree Crops | Blackberry | Conventional data pair available | 4.88 |
| Fruit & Tree Crops | Blackberry | No conventional data pair | 2.75 |
| Fruit & Tree Crops | Blueberry | Conventional data pair available | 4.26 |
| Fruit & Tree Crops | Blueberry | No conventional data pair | 0.64 |
| Fruit & Tree Crops | Cantaloupe | Conventional data pair available | 10.86 |
| Fruit & Tree Crops | Cantaloupe | No conventional data pair | 8.19 |
| Fruit & Tree Crops | Cherry, sweet | Conventional data pair available | 7.41 |
| Fruit & Tree Crops | Cherry, sweet | No conventional data pair | 3.46 |
| Fruit & Tree Crops | Fig | Conventional data pair available | 4.19 |
| Fruit & Tree Crops | Fig | No conventional data pair | 4.48 |
| Fruit & Tree Crops | Grapefruit | Conventional data pair available | 25.67 |
| Fruit & Tree Crops | Grapefruit | No conventional data pair | 10.85 |
| Fruit & Tree Crops | Grapes | Conventional data pair available | 5.54 |
| Fruit & Tree Crops | Grapes | No conventional data pair | 2.71 |
| Fruit & Tree Crops | Lemon | Conventional data pair available | 28.45 |
| Fruit & Tree Crops | Orange | Conventional data pair available | 18.68 |
| Fruit & Tree Crops | Orange | No conventional data pair | 3.35 |
| Fruit & Tree Crops | Peach | Conventional data pair available | 9.86 |
| Fruit & Tree Crops | Peach | No conventional data pair | 9.80 |
| Fruit & Tree Crops | Pear | Conventional data pair available | 13.93 |
| Fruit & Tree Crops | Pear | No conventional data pair | 11.98 |
| Fruit & Tree Crops | Plum/Prune | Conventional data pair available | 5.80 |
| Fruit & Tree Crops | Plum/Prune | No conventional data pair | 6.28 |
| Fruit & Tree Crops | Raspberry | Conventional data pair available | 7.08 |
| Fruit & Tree Crops | Raspberry | No conventional data pair | 1.21 |
| Fruit & Tree Crops | Strawberry | Conventional data pair available | 10.49 |
| Fruit & Tree Crops | Strawberry | No conventional data pair | 11.77 |
| Fruit & Tree Crops | Watermelon | Conventional data pair available | 15.46 |
| Fruit & Tree Crops | Watermelon | No conventional data pair | 13.26 |
| Vegetables | Artichoke | Conventional data pair available | 15.61 |
| Vegetables | Artichoke | No conventional data pair | 12.18 |
| Vegetables | Bean, snap | Conventional data pair available | 5.19 |
| Vegetables | Bean, snap | No conventional data pair | 5.15 |
| Vegetables | Broccoli | Conventional data pair available | 16.20 |
| Vegetables | Broccoli | No conventional data pair | 6.14 |
| Vegetables | Carrot | Conventional data pair available | 14.71 |
| Vegetables | Carrot | No conventional data pair | 10.33 |
| Vegetables | Cauliflower | Conventional data pair available | 12.05 |
| Vegetables | Cauliflower | No conventional data pair | 5.46 |
| Vegetables | Celery | Conventional data pair available | 50.76 |
| Vegetables | Celery | No conventional data pair | 10.49 |
| Vegetables | Garlic | Conventional data pair available | 5.03 |
| Vegetables | Garlic | No conventional data pair | 3.07 |
| Vegetables | Lettuce (all) | Conventional data pair available | 14.73 |
| Vegetables | Lettuce (all) | No conventional data pair | 9.37 |
| Vegetables | Maize (sweet) | Conventional data pair available | 7.85 |
| Vegetables | Maize (sweet) | No conventional data pair | 4.02 |
| Vegetables | Onion | Conventional data pair available | 20.26 |
| Vegetables | Onion | No conventional data pair | 11.48 |
| Vegetables | Pea | Conventional data pair available | 7.21 |
| Vegetables | Pea | No conventional data pair | 15.10 |
| Vegetables | Pepper, bell | Conventional data pair available | 16.41 |
| Vegetables | Pepper, bell | No conventional data pair | 8.13 |
| Vegetables | Potato | Conventional data pair available | 16.81 |
| Vegetables | Potato | No conventional data pair | 6.14 |
| Vegetables | Spinach | Conventional data pair available | 7.10 |
| Vegetables | Spinach | No conventional data pair | 5.61 |
| Vegetables | Squash | Conventional data pair available | 12.66 |
| Vegetables | Squash | No conventional data pair | 9.41 |
| Vegetables | Sweet potato | Conventional data pair available | 20.31 |
| Vegetables | Sweet potato | No conventional data pair | 8.97 |
| Vegetables | Tomato | Conventional data pair available | 16.36 |
| Vegetables | Tomato | No conventional data pair | 11.65 |

# Field & Forage Crops

## Median and weighted mean yield ratios for field & forage crops (Manuscript Fig 1).

|  | Crop | NumStates | MedianRY | RelYield |
| --- | --- | --- | --- | --- |
| 5 | Barley | 18 | 0.65 | 0.76 |
| 19 | Dry edible bean | 11 | 0.62 | 0.74 |
| 25 | Hay & alfalfa mix | 28 | 0.94 | 1.00 |
| 26 | Hay (all) | 39 | 1.14 | 1.12 |
| 27 | Haylage | 15 | 1.89 | 1.64 |
| 28 | Hay (other) | 31 | 1.12 | 1.20 |
| 34 | Maize (grain) | 26 | 0.66 | 0.65 |
| 35 | Maize (silage) | 23 | 0.77 | 0.78 |
| 37 | Oat | 21 | 0.74 | 0.80 |
| 53 | Soybean | 19 | 0.69 | 0.68 |
| 62 | Wheat (all) | 29 | 0.63 | 0.66 |
| 64 | Wheat (spring) | 7 | 0.71 | 0.70 |
| 65 | Wheat (winter) | 25 | 0.63 | 0.66 |

## Dry edible bean production data by state

| State | Organic farms | Organic hectares | Organic yield (ton/ha) | Total hectares | Total yield (ton/ha) | Yield ratio |
| --- | --- | --- | --- | --- | --- | --- |
| Arizona | 6 | 2967 | 2.98 | 4413 | 2.17 | 1.376 |
| New York | 8 | 223 | 1.98 | 3117 | 1.67 | 1.183 |
| Idaho | 10 | 143 | 2.23 | 50202 | 2.02 | 1.107 |
| Michigan | 49 | 2441 | 1.90 | 99312 | 2.17 | 0.878 |
| North Dakota | 5 | 391 | 1.08 | 248988 | 1.60 | 0.673 |
| Minnesota | 8 | 296 | 1.27 | 59919 | 2.18 | 0.582 |
| California | 6 | 298 | 1.34 | 19231 | 2.45 | 0.549 |
| Nebraska | 4 | 174 | 1.53 | 61538 | 2.80 | 0.546 |
| Montana | 8 | 445 | 0.88 | 14980 | 1.83 | 0.482 |
| Colorado | 14 | 474 | 0.96 | 17814 | 2.13 | 0.450 |
| Wisconsin | 8 | 569 | 1.13 | 3198 | 2.78 | 0.407 |

The median yield ratio for dry edible bean was 0.62, substantially less than the weighted mean yield ratio of 0.74. Of the 11 states where both organic and conventional dry edible bean were grown in 2014, only 3 reported organic yields greater than conventional yield (Table 2). Arizona had the greatest organic dry edible bean area (35% of the total) and also had the greatest yield ratio (1.38). The top four states for conventional dry bean area (Michigan, North Dakota, Minnesota, and Nebraska) had much lower organic yield compared to conventional (average weighted yield ratio of 0.67).

It is unclear why such a difference between states was observed with organic to conventional yield ratio in dry edible bean. Since it is a legume, nitrogen deficiency should play a minimal role in contrast to many other organic crops, as long as the seed is inoculated with the appropriate rhizobium species. Idaho and Colorado represent relatively similar growing environments with respect to dry edible bean production, and conventional yields were similar between these two states (Table 2). Even though conventional yields were similar, organic to conventional yield ratios of 1.11 and 0.45, were observed in Idaho and Colorado, respectively, because organic dry bean yield was much lower in Colorado. Regardless of the reason for the difference, these results suggest that dry edible bean has potential to be grown under organic management practices without sacrificing yield, but that potential is not being met in most organic production areas.

## Barley production data by state

| State | Organic farms | Organic hectares | Organic yield (ton/ha) | Total hectares | Total yield (ton/ha) | Yield ratio |
| --- | --- | --- | --- | --- | --- | --- |
| Washington | 13 | 211 | 3.67 | 42510 | 3.23 | 1.137 |
| Oregon | 31 | 3330 | 2.75 | 12146 | 2.69 | 1.022 |
| California | 21 | 1495 | 3.82 | 10121 | 3.92 | 0.973 |
| North Dakota | 7 | 709 | 3.23 | 216599 | 3.60 | 0.897 |
| Wisconsin | 122 | 1705 | 2.21 | 6478 | 2.53 | 0.874 |
| South Dakota | 7 | 260 | 2.27 | 6883 | 2.80 | 0.811 |
| Minnesota | 58 | 1319 | 2.25 | 24291 | 2.80 | 0.804 |
| New York | 29 | 454 | 2.00 | 3239 | 2.53 | 0.793 |
| Michigan | 9 | 54 | 2.05 | 2834 | 2.85 | 0.718 |
| Pennsylvania | 27 | 155 | 2.71 | 20243 | 3.82 | 0.710 |
| Colorado | 4 | 422 | 4.22 | 21862 | 6.66 | 0.633 |
| Arizona | 3 | 221 | 4.01 | 12955 | 6.72 | 0.597 |
| Virginia | 8 | 146 | 2.48 | 11336 | 4.25 | 0.583 |
| Maine | 9 | 98 | 2.08 | 4858 | 3.65 | 0.570 |
| Idaho | 46 | 5105 | 2.61 | 206478 | 5.05 | 0.516 |
| North Carolina | 7 | 145 | 1.85 | 6073 | 3.82 | 0.484 |
| Maryland | 9 | 182 | 1.85 | 18219 | 4.14 | 0.448 |
| Montana | 17 | 840 | 1.39 | 311741 | 3.12 | 0.447 |

The median yield ratio for barley was substantially less than the weighted mean yield ratio (Table Xbarley). Of the 18 states reporting both conventional and organic barley yield, only 2 (Washington and Oregon) had a yield ratio greater than 1. In general, the states with the most organic farms reporting had yield ratios closer to 1 compared to states with fewer organic farms. The most notable exception to this trend was Idaho. Idaho had the largest area devoted to organic barley production (5105 hectares) and the greatest number of organic barley farmers responding to the survey (46), yet had among the lowest yield ratios, with organic barley yielding 52% of conventional barley. Idaho was the second leading producer of conventional barley in terms of area in 2014, producing over 200,000 hectares.

## Haylage production data by state

| State | Organic farms | Organic hectares | Organic yield (ton/ha) | Total hectares | Total yield (ton/ha) | Yield ratio |
| --- | --- | --- | --- | --- | --- | --- |
| Texas | 6 | 413 | 18.2 | 2244939 | 4.9 | 3.691 |
| South Dakota | 6 | 626 | 12.5 | 1327935 | 4.7 | 2.675 |
| Missouri | 6 | 205 | 10.0 | 1445344 | 4.6 | 2.185 |
| Michigan | 18 | 332 | 14.2 | 489879 | 6.9 | 2.059 |
| Vermont | 95 | 6174 | 11.3 | 117409 | 5.8 | 1.942 |
| Washington | 45 | 2251 | 16.7 | 376518 | 8.7 | 1.923 |
| Ohio | 89 | 1847 | 12.7 | 417004 | 6.6 | 1.916 |
| Idaho | 4 | 244 | 15.3 | 591093 | 8.5 | 1.807 |
| California | 50 | 5257 | 22.8 | 631579 | 12.7 | 1.786 |
| Minnesota | 67 | 2660 | 9.7 | 894737 | 5.7 | 1.684 |
| Illinois | 7 | 188 | 13.2 | 218623 | 7.9 | 1.669 |
| Pennsylvania | 150 | 4007 | 9.2 | 696356 | 5.9 | 1.551 |
| Iowa | 61 | 1166 | 11.6 | 493927 | 7.6 | 1.522 |
| Wisconsin | 232 | 8950 | 12.0 | 1093117 | 7.9 | 1.520 |
| New York | 193 | 8299 | 8.5 | 729960 | 5.9 | 1.447 |

Of the 15 states reporting both organic and conventional haylage yields, all reported greater organic haylage yields compared to conventional (Table Xhay). The organic to conventional haylage yield ratios ranged from 1.4 to 3.7. States with the greatest number of organic farms and organic area tended to have lower yield ratios, which resulted in a weighted mean substantially less than the median yield ratio for haylage (Fig 1).

# Fruits and Vegetables

## Median and weighted mean yield ratios for fruit & vegetable crops (Manuscript Fig 2).

| Crop | NumStates | MedianRY | RelYield |
| --- | --- | --- | --- |
| Apple | 16 | 0.29 | 0.60 |
| Blueberry | 9 | 0.62 | 0.56 |
| Grapes | 7 | 0.61 | 0.50 |
| Maize (sweet) | 16 | 0.73 | 0.77 |
| Onion | 7 | 0.41 | 0.40 |
| Peach | 7 | 1.07 | 0.67 |
| Pepper, bell | 7 | 0.46 | 0.48 |
| Potato | 20 | 0.26 | 0.38 |
| Snap bean | 11 | 0.75 | 0.75 |
| Squash | 11 | 0.72 | 0.74 |
| Tomato | 15 | 0.58 | 0.50 |
| Watermelon | 9 | 0.61 | 0.32 |

## Peach production data by state

| State | Organic farms | Organic hectares | Organic yield (ton/ha) | Total hectares | Total yield (ton/ha) | Yield ratio |
| --- | --- | --- | --- | --- | --- | --- |
| Arkansas | 3 | 1 | 13.4 | 263 | 2.2 | 6.000 |
| Washington | 36 | 109 | 23.0 | 972 | 11.7 | 1.971 |
| Georgia | 4 | 0 | 9.0 | 4413 | 7.3 | 1.227 |
| Texas | 7 | 1 | 3.4 | 1255 | 2.8 | 1.220 |
| California | 142 | 668 | 17.2 | 17814 | 31.6 | 0.545 |
| Utah | 4 | 0 | 2.2 | 526 | 11.2 | 0.200 |
| Pennsylvania | 9 | 6 | 0.8 | 1619 | 8.4 | 0.096 |

Median peach yield ratio was greater than 1, while the weighted mean was less than 1. The difference between the median and mean for the peach yield data was influenced by many states with very few organic acres in production (Table 3). Four of the seven states reporting both organic and conventional peach yield data had yield ratios greater than 1, but these states produced relatively few organic hectares (and had relatively few organic farms). Arkansas, for example, had a yield ratio of 6.0, but only 3 organic farms producing a total of 1 organic hectare. Georgia, the state with the second highest area of conventional peach production, had less than 1 organic hectare represented in the comparison. Washington and California, the two states with the largest number of organic farms and area, had yield ratios of 1.97 and 0.55, respectively. We were unable to find an explanation for the large difference between the yield ratios from Washington and California. It may relate to the ratio of processing to fresh market production in each state, and within each system within a state, since production issues like early frost and pest management can be less problematic for processing than for the fresh market.

## Apple production data by state

| State | Organic farms | Organic hectares | Organic yield (ton/ha) | Total hectares | Total yield (ton/ha) | Yield ratio |
| --- | --- | --- | --- | --- | --- | --- |
| Colorado | 17 | 79 | 20.3 | 486 | 8.3 | 2.440 |
| Ohio | 3 | 0 | 26.9 | 1457 | 13.7 | 1.966 |
| Utah | 5 | 5 | 21.9 | 526 | 19.8 | 1.103 |
| California | 245 | 1227 | 14.4 | 6073 | 17.9 | 0.806 |
| Washington | 221 | 4523 | 43.0 | 59919 | 55.2 | 0.778 |
| New York | 24 | 65 | 20.1 | 16194 | 36.3 | 0.554 |
| Oregon | 71 | 111 | 16.9 | 2065 | 34.0 | 0.498 |
| Minnesota | 28 | 46 | 4.3 | 1053 | 10.8 | 0.402 |
| Maine | 27 | 58 | 6.1 | 1093 | 15.8 | 0.390 |
| Wisconsin | 47 | 43 | 4.6 | 1619 | 15.1 | 0.305 |
| North Carolina | 11 | 5 | 4.1 | 2429 | 23.3 | 0.178 |
| Massachusetts | 6 | 9 | 2.5 | 1255 | 15.6 | 0.161 |
| Maryland | 7 | 4 | 4.1 | 729 | 25.8 | 0.158 |
| Virginia | 9 | 7 | 2.6 | 4211 | 21.0 | 0.124 |
| New Hampshire | 11 | 4 | 1.2 | 526 | 14.6 | 0.083 |
| Idaho | 9 | 2 | 2.0 | 972 | 29.5 | 0.067 |

The median yield ratio for apple was much lower than the weighted mean (Fig 2). The median was influenced by a large number of states with less than 10 hectares of organic apples that had organic yields less than 20% of conventional (Table Xapple). The two states with the greatest organic production both in terms of farms and area (California and Washington) had organic yield that was 81% and 78% of conventional, respectively, compared to the weighted mean of 60% of conventional for all states reporting both conventional and organic apple production.

## Potato production data by state

| State | Organic farms | Organic hectares | Organic yield (Ton/ha) | Total hectares | Total yield (ton/ha) | Yield ratio |
| --- | --- | --- | --- | --- | --- | --- |
| Washington | 60 | 528 | 60.6 | 66802 | 68.9 | 0.880 |
| Colorado | 15 | 1426 | 33.5 | 24211 | 43.4 | 0.772 |
| Wisconsin | 69 | 93 | 33.2 | 25911 | 45.9 | 0.724 |
| Maine | 68 | 166 | 20.0 | 20445 | 32.5 | 0.617 |
| Idaho | 13 | 31 | 26.1 | 129555 | 46.5 | 0.561 |
| California | 82 | 1161 | 28.6 | 13401 | 52.6 | 0.543 |
| Oregon | 44 | 1257 | 31.1 | 15749 | 64.9 | 0.478 |
| Texas | 14 | 11 | 17.8 | 8340 | 37.5 | 0.475 |
| Pennsylvania | 44 | 29 | 10.3 | 2105 | 30.8 | 0.335 |
| Massachusetts | 31 | 9 | 10.3 | 1457 | 31.9 | 0.322 |
| Minnesota | 33 | 4 | 12.9 | 16599 | 44.8 | 0.289 |
| Ohio | 42 | 8 | 9.1 | 607 | 31.4 | 0.289 |
| Michigan | 17 | 2 | 10.5 | 17206 | 41.4 | 0.254 |
| New York | 58 | 27 | 5.6 | 6397 | 30.8 | 0.182 |
| Illinois | 19 | 3 | 8.0 | 2591 | 46.5 | 0.172 |
| Kansas | 11 | 2 | 6.0 | 1660 | 38.1 | 0.157 |
| Maryland | 14 | 4 | 5.4 | 931 | 42.6 | 0.126 |
| North Carolina | 36 | 13 | 2.8 | 5466 | 23.5 | 0.120 |
| Virginia | 17 | 10 | 2.9 | 1822 | 28.0 | 0.105 |
| Florida | 5 | 2 | 1.6 | 11862 | 26.9 | 0.058 |

Potato may be among the most difficult crops to grow organically relative to conventional. Organic potato yield was only 38% of conventional potato yield in our analysis, and the median was even lower, at 26% of conventional yield (Fig 2). None of the 20 states in our analysis reported organic yields greater than conventional, and only one state (Washington) reported organic potato yield more than 80% of conventional (Table Xpotato).

## Grape production data by state

| State | Organic farms | Organic hectares | Organic yield (Ton/ha) | Total hectares | Total yield (ton/ha) | Yield ratio |
| --- | --- | --- | --- | --- | --- | --- |
| Oregon | 55 | 472 | 6.4 | 7692 | 6.8 | 0.931 |
| Washington | 71 | 946 | 13.0 | 29150 | 15.9 | 0.816 |
| New York | 22 | 74 | 8.8 | 14980 | 11.4 | 0.770 |
| California | 533 | 9443 | 8.4 | 350202 | 17.7 | 0.474 |
| North Carolina | 4 | 12 | 1.4 | 931 | 5.8 | 0.238 |
| Georgia | 16 | 5 | 0.7 | 648 | 5.6 | 0.124 |
| Michigan | 9 | 9 | 0.2 | 5506 | 10.4 | 0.022 |

Of the seven states that reported both organic and conventional grape yields, none had yield ratios greater than 1. The grape yield ratio may be skewed somewhat in California (the largest grape producer for both organic and conventional) because organic wine grapes are often produced in low yielding coastal areas, while the conventional data includes the higher yielding Central Valley of California.

# Weighted and unweighted slope parameters regressing yield ratio onto conventional yield (Manuscript Fig 4)

| Crop | Unweighted slope | SE | P | Rsq | Weighted slope | SE | P | Rsq |
| --- | --- | --- | --- | --- | --- | --- | --- | --- |
| Apple | -0.005 | 0.024 | 0.836 | 0.003 | 0.007 | 0.009 | 0.468 | 0.038 |
| Barley | -0.093 | 0.052 | 0.092 | 0.167 | -0.166 | 0.052 | 0.005 | 0.393 |
| Blueberry | -0.017 | 0.035 | 0.640 | 0.033 | -0.031 | 0.033 | 0.373 | 0.114 |
| Dry edible bean | -0.462 | 0.320 | 0.183 | 0.188 | -0.508 | 0.396 | 0.231 | 0.155 |
| Grapes | 0.078 | 0.121 | 0.546 | 0.077 | 0.001 | 0.061 | 0.982 | 0.000 |
| Hay & alfalfa mix | -0.056 | 0.015 | 0.001 | 0.354 | -0.065 | 0.016 | 0.000 | 0.393 |
| Hay (all) | -0.076 | 0.020 | 0.000 | 0.285 | -0.083 | 0.016 | 0.000 | 0.425 |
| Haylage | -0.045 | 0.030 | 0.159 | 0.146 | 0.002 | 0.023 | 0.921 | 0.001 |
| Hay (other) | -0.199 | 0.060 | 0.002 | 0.275 | -0.203 | 0.035 | 0.000 | 0.530 |
| Maize (grain) | -0.077 | 0.027 | 0.008 | 0.258 | -0.038 | 0.025 | 0.136 | 0.090 |
| Maize (silage) | 0.000 | 0.004 | 0.911 | 0.001 | 0.004 | 0.004 | 0.393 | 0.035 |
| Maize (sweet) | -0.022 | 0.053 | 0.687 | 0.012 | 0.004 | 0.033 | 0.894 | 0.001 |
| Oat | -0.108 | 0.153 | 0.490 | 0.025 | -0.028 | 0.117 | 0.816 | 0.003 |
| Onion | 0.014 | 0.014 | 0.364 | 0.166 | 0.020 | 0.017 | 0.309 | 0.204 |
| Peach | -0.042 | 0.060 | 0.517 | 0.088 | -0.029 | 0.028 | 0.351 | 0.175 |
| Pepper, bell | 0.038 | 0.039 | 0.371 | 0.162 | 0.025 | 0.022 | 0.310 | 0.203 |
| Potato | 0.038 | 0.012 | 0.005 | 0.362 | 0.030 | 0.009 | 0.003 | 0.389 |
| Snap bean | -0.021 | 0.085 | 0.810 | 0.007 | 0.023 | 0.060 | 0.709 | 0.016 |
| Soybean | 0.091 | 0.063 | 0.169 | 0.108 | 0.173 | 0.045 | 0.001 | 0.459 |
| Squash | -0.069 | 0.031 | 0.052 | 0.359 | -0.054 | 0.033 | 0.132 | 0.234 |
| Tomato | -0.009 | 0.012 | 0.473 | 0.040 | -0.010 | 0.011 | 0.402 | 0.055 |
| Watermelon | 0.006 | 0.022 | 0.797 | 0.010 | -0.003 | 0.014 | 0.848 | 0.006 |
| Wheat (all) | -0.134 | 0.042 | 0.004 | 0.274 | -0.117 | 0.041 | 0.009 | 0.229 |
| Wheat (spring) | -0.098 | 0.106 | 0.397 | 0.146 | -0.165 | 0.143 | 0.300 | 0.211 |
| Wheat (winter) | -0.166 | 0.062 | 0.014 | 0.236 | -0.135 | 0.054 | 0.021 | 0.212 |

# Data summarized by crop

| Group | Crop | Number of states | Organic farms | Organic hectares | Total hectares | Yield ratio |
| --- | --- | --- | --- | --- | --- | --- |
| Field Crops | Barley | 18 | 427 | 16852 | 938866 | 0.76 |
| Field Crops | Dry edible bean | 11 | 126 | 8421 | 582713 | 0.74 |
| Field Crops | Dry edible pea | 2 | 15 | 1529 | 307287 | 0.60 |
| Field Crops | Lentil | 1 | 10 | 325 | 48178 | 0.53 |
| Field Crops | Maize (grain) | 26 | 2478 | 62918 | 32153441 | 0.65 |
| Field Crops | Oat | 21 | 974 | 17147 | 360729 | 0.80 |
| Field Crops | Proso millet | 2 | 29 | 4132 | 146154 | 0.73 |
| Field Crops | Rice | 2 | 80 | 8762 | 234008 | 0.60 |
| Field Crops | Safflower | 1 | 4 | 662 | 7287 | 0.52 |
| Field Crops | Sorghum (grain) | 3 | 26 | 1715 | 2033603 | 0.83 |
| Field Crops | Soybean | 19 | 1418 | 39694 | 29832794 | 0.68 |
| Field Crops | Sunflower | 3 | 16 | 1741 | 488057 | 0.81 |
| Field Crops | Wheat (all) | 29 | 1047 | 106285 | 17912955 | 0.66 |
| Field Crops | Wheat (durum) | 3 | 21 | 3572 | 506073 | 0.58 |
| Field Crops | Wheat (spring) | 7 | 211 | 30683 | 5298381 | 0.70 |
| Field Crops | Wheat (winter) | 25 | 679 | 59008 | 10283806 | 0.66 |
| Forage Crops | Hay & alfalfa mix | 28 | 2051 | 100881 | 6880567 | 1.00 |
| Forage Crops | Hay (all) | 39 | 3689 | 172787 | 20514575 | 1.12 |
| Forage Crops | Haylage | 15 | 1029 | 42619 | 11768421 | 1.64 |
| Forage Crops | Hay (other) | 31 | 1707 | 63284 | 11824696 | 1.20 |
| Forage Crops | Maize (silage) | 23 | 946 | 13952 | 2038462 | 0.78 |
| Forage Crops | Sorghum (silage) | 4 | 23 | 3858 | 58300 | 0.75 |
| Fruit & Tree Crops | Almond | 1 | 99 | 2495 | 352227 | 0.57 |
| Fruit & Tree Crops | Apple | 16 | 741 | 6189 | 100607 | 0.60 |
| Fruit & Tree Crops | Avocado | 3 | 371 | 1547 | 24818 | 0.94 |
| Fruit & Tree Crops | Blackberry | 1 | 19 | 24 | 2470 | 0.58 |
| Fruit & Tree Crops | Blueberry | 9 | 316 | 1333 | 24668 | 0.56 |
| Fruit & Tree Crops | Blueberry (wild) | 1 | 46 | 161 | 9231 | 0.22 |
| Fruit & Tree Crops | Cantaloupe | 6 | 142 | 327 | 22733 | 0.73 |
| Fruit & Tree Crops | Cherry | 2 | 118 | 692 | 27530 | 0.84 |
| Fruit & Tree Crops | Cherry (tart) | 1 | 4 | 1 | 263 | 0.17 |
| Fruit & Tree Crops | Cranberry | 2 | 20 | 107 | 13401 | 0.36 |
| Fruit & Tree Crops | Dates | 1 | 23 | 286 | 3320 | 0.90 |
| Fruit & Tree Crops | Fig | 1 | 86 | 494 | 2834 | 0.41 |
| Fruit & Tree Crops | Grapefruit | 3 | 130 | 543 | 28138 | 0.66 |
| Fruit & Tree Crops | Grapes | 7 | 710 | 10960 | 409109 | 0.50 |
| Fruit & Tree Crops | Hazelnut | 1 | 7 | 74 | 12146 | 0.61 |
| Fruit & Tree Crops | Lemon | 1 | 195 | 857 | 18623 | 0.78 |
| Fruit & Tree Crops | Orange | 2 | 298 | 1977 | 67206 | 0.68 |
| Fruit & Tree Crops | Peach | 7 | 205 | 785 | 26862 | 0.67 |
| Fruit & Tree Crops | Pear | 5 | 246 | 1083 | 19636 | 0.54 |
| Fruit & Tree Crops | Plum/prune | 2 | 159 | 974 | 7814 | 0.51 |
| Fruit & Tree Crops | Raspberry | 3 | 133 | 185 | 7996 | 0.57 |
| Fruit & Tree Crops | Strawberry | 5 | 267 | 1087 | 17814 | 0.48 |
| Fruit & Tree Crops | Walnut | 1 | 195 | 2326 | 117409 | 0.69 |
| Fruit & Tree Crops | Watermelon | 9 | 133 | 249 | 34919 | 0.32 |
| Other | Hops | 1 | 5 | 93 | 11683 | 0.60 |
| Vegetables | Artichoke | 1 | 28 | 41 | 2955 | 1.07 |
| Vegetables | Broccoli | 1 | 150 | 2934 | 49393 | 0.91 |
| Vegetables | Cabbage | 6 | 369 | 292 | 12777 | 0.56 |
| Vegetables | Carrot | 4 | 256 | 2928 | 29231 | 0.94 |
| Vegetables | Cauliflower | 2 | 90 | 610 | 13915 | 0.71 |
| Vegetables | Celery | 1 | 67 | 817 | 11012 | 0.71 |
| Vegetables | Garlic | 3 | 153 | 142 | 9636 | 0.54 |
| Vegetables | Lettuce (all) | 2 | 174 | 12643 | 105668 | 0.29 |
| Vegetables | Maize (sweet) | 16 | 315 | 4630 | 67980 | 0.77 |
| Vegetables | Onion | 7 | 167 | 420 | 36559 | 0.40 |
| Vegetables | Pea | 1 | 24 | 1334 | 7935 | 1.53 |
| Vegetables | Pepper, bell | 7 | 299 | 311 | 18826 | 0.48 |
| Vegetables | Potato | 20 | 692 | 4785 | 373117 | 0.38 |
| Vegetables | Snap bean | 11 | 318 | 832 | 60332 | 0.75 |
| Vegetables | Spinach | 3 | 24 | 340 | 4372 | 0.40 |
| Vegetables | Squash | 11 | 650 | 2042 | 15194 | 0.74 |
| Vegetables | Sweet potato | 4 | 77 | 2249 | 38785 | 0.89 |
| Vegetables | Tomato | 15 | 905 | 2881 | 153846 | 0.50 |

# Data sorted by crop and location

| Crop | Location | Number of organic farms | Organic hectares | Conventional hectares | Organic yield (t/ha) | Conventional yield (t/ha) | Yield ratio (Organic/Conventional) |
| --- | --- | --- | --- | --- | --- | --- | --- |
| Almond | California | 99 | 2495 | 352227 | 1.38 | 2.41 | 0.575 |
| Apple | California | 245 | 1227 | 6073 | 14.44 | 17.92 | 0.806 |
| Apple | Colorado | 17 | 79 | 486 | 20.27 | 8.31 | 2.440 |
| Apple | Idaho | 9 | 2 | 972 | 1.97 | 29.53 | 0.067 |
| Apple | Maine | 27 | 58 | 1093 | 6.14 | 15.76 | 0.390 |
| Apple | Maryland | 7 | 4 | 729 | 4.07 | 25.76 | 0.158 |
| Apple | Massachusetts | 6 | 9 | 1255 | 2.52 | 15.64 | 0.161 |
| Apple | Minnesota | 28 | 46 | 1053 | 4.33 | 10.77 | 0.402 |
| Apple | New Hampshire | 11 | 4 | 526 | 1.20 | 14.56 | 0.083 |
| Apple | New York | 24 | 65 | 16194 | 20.10 | 36.25 | 0.554 |
| Apple | North Carolina | 11 | 5 | 2429 | 4.14 | 23.33 | 0.178 |
| Apple | Ohio | 3 | 0 | 1457 | 26.91 | 13.69 | 1.966 |
| Apple | Oregon | 71 | 111 | 2065 | 16.94 | 34.03 | 0.498 |
| Apple | Utah | 5 | 5 | 526 | 21.86 | 19.81 | 1.103 |
| Apple | Virginia | 9 | 7 | 4211 | 2.61 | 21.00 | 0.124 |
| Apple | Washington | 221 | 4523 | 59919 | 42.97 | 55.23 | 0.778 |
| Apple | Wisconsin | 47 | 43 | 1619 | 4.62 | 15.12 | 0.305 |
| Artichoke | California | 28 | 41 | 2955 | 15.61 | 14.56 | 1.072 |
| Avocado | California | 321 | 1479 | 21781 | 6.34 | 6.83 | 0.928 |
| Avocado | Florida | 10 | 53 | 2834 | 8.15 | 10.53 | 0.774 |
| Avocado | Hawaii | 40 | 15 | 202 | 2.60 | 2.46 | 1.055 |
| Barley | Arizona | 3 | 221 | 12955 | 4.01 | 6.72 | 0.597 |
| Barley | California | 21 | 1495 | 10121 | 3.82 | 3.92 | 0.973 |
| Barley | Colorado | 4 | 422 | 21862 | 4.22 | 6.66 | 0.633 |
| Barley | Idaho | 46 | 5105 | 206478 | 2.61 | 5.05 | 0.516 |
| Barley | Maine | 9 | 98 | 4858 | 2.08 | 3.65 | 0.570 |
| Barley | Maryland | 9 | 182 | 18219 | 1.85 | 4.14 | 0.448 |
| Barley | Michigan | 9 | 54 | 2834 | 2.05 | 2.85 | 0.718 |
| Barley | Minnesota | 58 | 1319 | 24291 | 2.25 | 2.80 | 0.804 |
| Barley | Montana | 17 | 840 | 311741 | 1.39 | 3.12 | 0.447 |
| Barley | New York | 29 | 454 | 3239 | 2.00 | 2.53 | 0.793 |
| Barley | North Carolina | 7 | 145 | 6073 | 1.85 | 3.82 | 0.484 |
| Barley | North Dakota | 7 | 709 | 216599 | 3.23 | 3.60 | 0.897 |
| Barley | Oregon | 31 | 3330 | 12146 | 2.75 | 2.69 | 1.022 |
| Barley | Pennsylvania | 27 | 155 | 20243 | 2.71 | 3.82 | 0.710 |
| Barley | South Dakota | 7 | 260 | 6883 | 2.27 | 2.80 | 0.811 |
| Barley | Virginia | 8 | 146 | 11336 | 2.48 | 4.25 | 0.583 |
| Barley | Washington | 13 | 211 | 42510 | 3.67 | 3.23 | 1.137 |
| Barley | Wisconsin | 122 | 1705 | 6478 | 2.21 | 2.53 | 0.874 |
| Blackberry | Oregon | 19 | 24 | 2470 | 4.88 | 8.35 | 0.584 |
| Blueberry | Arkansas | 8 | 2 | 93 | 1.00 | 3.02 | 0.331 |
| Blueberry | California | 63 | 381 | 1943 | 9.91 | 13.32 | 0.744 |
| Blueberry | Florida | 39 | 121 | 1741 | 4.18 | 5.31 | 0.787 |
| Blueberry | Georgia | 26 | 106 | 6721 | 3.88 | 6.61 | 0.587 |
| Blueberry | New Jersey | 10 | 53 | 3765 | 4.38 | 6.70 | 0.654 |
| Blueberry | New York | 28 | 36 | 283 | 2.39 | 2.62 | 0.909 |
| Blueberry | North Carolina | 28 | 37 | 2794 | 3.74 | 7.92 | 0.473 |
| Blueberry | Oregon | 58 | 88 | 3644 | 4.23 | 10.86 | 0.389 |
| Blueberry | Washington | 56 | 508 | 3684 | 4.67 | 11.64 | 0.401 |
| Blueberry (wild) | Maine | 46 | 161 | 9231 | 1.12 | 5.13 | 0.219 |
| Broccoli | California | 150 | 2934 | 49393 | 16.20 | 17.88 | 0.907 |
| Cabbage | Arizona | 5 | 29 | 1498 | 37.20 | 57.69 | 0.645 |
| Cabbage | California | 129 | 194 | 6640 | 25.46 | 47.03 | 0.541 |
| Cabbage | Ohio | 54 | 9 | 567 | 16.07 | 39.19 | 0.410 |
| Cabbage | Pennsylvania | 43 | 20 | 389 | 15.80 | 24.73 | 0.639 |
| Cabbage | Texas | 15 | 6 | 2510 | 10.23 | 27.99 | 0.366 |
| Cabbage | Wisconsin | 123 | 34 | 1174 | 18.42 | 27.99 | 0.658 |
| Cantaloupe | California | 93 | 317 | 18826 | 25.92 | 29.37 | 0.883 |
| Cantaloupe | Georgia | 12 | 4 | 1336 | 9.03 | 13.44 | 0.672 |
| Cantaloupe | Indiana | 7 | 0 | 729 | 2.69 | 24.64 | 0.109 |
| Cantaloupe | Maryland | 9 | 2 | 243 | 8.17 | 14.37 | 0.569 |
| Cantaloupe | Pennsylvania | 12 | 1 | 486 | 13.92 | 13.44 | 1.036 |
| Cantaloupe | Texas | 9 | 2 | 1113 | 5.43 | 14.82 | 0.366 |
| Carrot | California | 162 | 2906 | 26518 | 23.00 | 35.83 | 0.642 |
| Carrot | Michigan | 15 | 4 | 648 | 11.20 | 34.71 | 0.323 |
| Carrot | Texas | 19 | 8 | 567 | 8.52 | 30.23 | 0.282 |
| Carrot | Wisconsin | 60 | 11 | 1498 | 16.11 | 3.24 | 4.970 |
| Cauliflower | California | 76 | 609 | 13725 | 15.20 | 20.72 | 0.734 |
| Cauliflower | New York | 14 | 2 | 190 | 8.90 | 14.53 | 0.613 |
| Celery | California | 67 | 817 | 11012 | 50.76 | 71.67 | 0.708 |
| Cherry | California | 38 | 121 | 13360 | 1.84 | 2.26 | 0.812 |
| Cherry | Washington | 80 | 570 | 14170 | 12.99 | 15.16 | 0.857 |
| Cherry (tart) | Oregon | 4 | 1 | 263 | 0.72 | 4.14 | 0.173 |
| Cranberry | Massachusetts | 11 | 35 | 5020 | 8.55 | 18.69 | 0.457 |
| Cranberry | Wisconsin | 9 | 72 | 8381 | 7.44 | 27.17 | 0.274 |
| Dates | California | 23 | 286 | 3320 | 7.17 | 7.93 | 0.904 |
| Dry edible bean | Arizona | 6 | 2967 | 4413 | 2.98 | 2.17 | 1.376 |
| Dry edible bean | California | 6 | 298 | 19231 | 1.34 | 2.45 | 0.549 |
| Dry edible bean | Colorado | 14 | 474 | 17814 | 0.96 | 2.13 | 0.450 |
| Dry edible bean | Idaho | 10 | 143 | 50202 | 2.23 | 2.02 | 1.107 |
| Dry edible bean | Michigan | 49 | 2441 | 99312 | 1.90 | 2.17 | 0.878 |
| Dry edible bean | Minnesota | 8 | 296 | 59919 | 1.27 | 2.18 | 0.582 |
| Dry edible bean | Montana | 8 | 445 | 14980 | 0.88 | 1.83 | 0.482 |
| Dry edible bean | Nebraska | 4 | 174 | 61538 | 1.53 | 2.80 | 0.546 |
| Dry edible bean | New York | 8 | 223 | 3117 | 1.98 | 1.67 | 1.183 |
| Dry edible bean | North Dakota | 5 | 391 | 248988 | 1.08 | 1.60 | 0.673 |
| Dry edible bean | Wisconsin | 8 | 569 | 3198 | 1.13 | 2.78 | 0.407 |
| Dry edible pea | Montana | 11 | 1288 | 204049 | 1.15 | 2.02 | 0.569 |
| Dry edible pea | North Dakota | 4 | 241 | 103239 | 1.63 | 2.39 | 0.682 |
| Fig | California | 86 | 494 | 2834 | 4.19 | 10.15 | 0.413 |
| Garlic | California | 96 | 130 | 9312 | 9.41 | 18.48 | 0.510 |
| Garlic | Nevada | 9 | 2 | 223 | 0.87 | 12.42 | 0.070 |
| Garlic | Oregon | 48 | 10 | 101 | 4.80 | 5.38 | 0.893 |
| Grapefruit | California | 116 | 444 | 3968 | 22.49 | 35.18 | 0.639 |
| Grapefruit | Florida | 7 | 23 | 17449 | 23.58 | 34.56 | 0.682 |
| Grapefruit | Texas | 7 | 76 | 6721 | 30.93 | 30.75 | 1.006 |
| Grapes | California | 533 | 9443 | 350202 | 8.38 | 17.67 | 0.474 |
| Grapes | Georgia | 16 | 5 | 648 | 0.69 | 5.60 | 0.124 |
| Grapes | Michigan | 9 | 9 | 5506 | 0.22 | 10.41 | 0.022 |
| Grapes | New York | 22 | 74 | 14980 | 8.76 | 11.38 | 0.770 |
| Grapes | North Carolina | 4 | 12 | 931 | 1.39 | 5.85 | 0.238 |
| Grapes | Oregon | 55 | 472 | 7692 | 6.36 | 6.83 | 0.931 |
| Grapes | Washington | 71 | 946 | 29150 | 12.99 | 15.92 | 0.816 |
| Hay & alfalfa mix | California | 77 | 8045 | 354251 | 9.43 | 14.56 | 0.648 |
| Hay & alfalfa mix | Colorado | 45 | 6378 | 299595 | 7.10 | 7.61 | 0.932 |
| Hay & alfalfa mix | Idaho | 82 | 15816 | 441296 | 5.02 | 8.73 | 0.574 |
| Hay & alfalfa mix | Illinois | 53 | 1160 | 109312 | 9.41 | 8.96 | 1.050 |
| Hay & alfalfa mix | Indiana | 124 | 1723 | 97166 | 8.60 | 8.96 | 0.960 |
| Hay & alfalfa mix | Iowa | 200 | 3503 | 327935 | 7.41 | 8.06 | 0.919 |
| Hay & alfalfa mix | Kentucky | 12 | 135 | 66802 | 5.40 | 7.61 | 0.709 |
| Hay & alfalfa mix | Maryland | 13 | 162 | 14170 | 7.93 | 8.51 | 0.932 |
| Hay & alfalfa mix | Massachusetts | 5 | 143 | 4049 | 3.74 | 4.03 | 0.928 |
| Hay & alfalfa mix | Michigan | 61 | 994 | 259109 | 5.55 | 6.49 | 0.855 |
| Hay & alfalfa mix | Minnesota | 185 | 6064 | 445344 | 7.86 | 6.49 | 1.210 |
| Hay & alfalfa mix | Missouri | 11 | 191 | 113360 | 4.41 | 5.60 | 0.788 |
| Hay & alfalfa mix | Montana | 34 | 5202 | 748988 | 3.65 | 4.70 | 0.776 |
| Hay & alfalfa mix | Nebraska | 60 | 3209 | 336032 | 9.29 | 9.18 | 1.012 |
| Hay & alfalfa mix | Nevada | 14 | 2051 | 113360 | 9.76 | 9.41 | 1.038 |
| Hay & alfalfa mix | New York | 102 | 2577 | 117409 | 5.89 | 5.82 | 1.012 |
| Hay & alfalfa mix | North Dakota | 39 | 6109 | 668016 | 4.48 | 4.70 | 0.952 |
| Hay & alfalfa mix | Ohio | 132 | 2803 | 125506 | 7.93 | 7.84 | 1.011 |
| Hay & alfalfa mix | Oregon | 63 | 9305 | 141700 | 8.94 | 9.85 | 0.907 |
| Hay & alfalfa mix | Pennsylvania | 166 | 3328 | 141700 | 6.79 | 6.27 | 1.082 |
| Hay & alfalfa mix | South Dakota | 34 | 4130 | 769231 | 4.77 | 1.81 | 2.630 |
| Hay & alfalfa mix | Texas | 9 | 1769 | 56680 | 13.48 | 9.85 | 1.368 |
| Hay & alfalfa mix | Utah | 11 | 823 | 210526 | 6.81 | 8.73 | 0.779 |
| Hay & alfalfa mix | Vermont | 25 | 837 | 14170 | 5.20 | 3.58 | 1.450 |
| Hay & alfalfa mix | Virginia | 8 | 121 | 30364 | 6.58 | 7.61 | 0.865 |
| Hay & alfalfa mix | Washington | 32 | 1107 | 170040 | 11.91 | 10.53 | 1.132 |
| Hay & alfalfa mix | Wisconsin | 444 | 11889 | 506073 | 8.17 | 7.39 | 1.106 |
| Hay & alfalfa mix | Wyoming | 10 | 1307 | 198381 | 9.20 | 18.86 | 0.488 |
| Hay (all) | Arizona | 7 | 330 | 121457 | 6.41 | 17.98 | 0.356 |
| Hay (all) | Arkansas | 3 | 33 | 495951 | 5.26 | 4.50 | 1.169 |
| Hay (all) | California | 142 | 12639 | 556680 | 8.00 | 12.03 | 0.665 |
| Hay (all) | Colorado | 59 | 8449 | 542510 | 6.16 | 5.96 | 1.034 |
| Hay (all) | Georgia | 4 | 20 | 234818 | 2.69 | 5.82 | 0.462 |
| Hay (all) | Idaho | 91 | 16391 | 562753 | 4.99 | 7.86 | 0.635 |
| Hay (all) | Illinois | 67 | 1450 | 210526 | 8.87 | 7.57 | 1.172 |
| Hay (all) | Indiana | 153 | 2291 | 242915 | 8.04 | 7.28 | 1.105 |
| Hay (all) | Iowa | 276 | 5345 | 467611 | 7.26 | 7.12 | 1.019 |
| Hay (all) | Kansas | 19 | 2175 | 931174 | 5.20 | 4.86 | 1.069 |
| Hay (all) | Kentucky | 33 | 527 | 917004 | 5.42 | 4.70 | 1.152 |
| Hay (all) | Maine | 92 | 2826 | 60729 | 4.90 | 3.47 | 1.413 |
| Hay (all) | Maryland | 27 | 640 | 78947 | 5.08 | 5.93 | 0.857 |
| Hay (all) | Massachusetts | 43 | 611 | 30364 | 5.26 | 3.85 | 1.366 |
| Hay (all) | Michigan | 103 | 2255 | 396761 | 5.11 | 5.87 | 0.870 |
| Hay (all) | Minnesota | 267 | 8984 | 773279 | 7.19 | 5.26 | 1.366 |
| Hay (all) | Missouri | 45 | 1246 | 1408907 | 5.13 | 4.57 | 1.123 |
| Hay (all) | Montana | 43 | 6847 | 1105263 | 3.47 | 4.41 | 0.787 |
| Hay (all) | Nebraska | 65 | 3415 | 1044534 | 9.20 | 5.24 | 1.756 |
| Hay (all) | Nevada | 14 | 2051 | 174089 | 9.76 | 7.37 | 1.325 |
| Hay (all) | New Hampshire | 20 | 469 | 21862 | 2.06 | 3.90 | 0.529 |
| Hay (all) | New Jersey | 17 | 155 | 42915 | 2.42 | 5.51 | 0.439 |
| Hay (all) | New Mexico | 12 | 760 | 123482 | 7.28 | 8.80 | 0.827 |
| Hay (all) | New York | 346 | 14740 | 554656 | 5.98 | 4.41 | 1.355 |
| Hay (all) | North Carolina | 10 | 219 | 336032 | 4.79 | 5.38 | 0.892 |
| Hay (all) | North Dakota | 42 | 6998 | 1093117 | 4.77 | 4.52 | 1.054 |
| Hay (all) | Ohio | 250 | 5235 | 388664 | 6.94 | 6.32 | 1.099 |
| Hay (all) | Oregon | 103 | 14052 | 417004 | 7.57 | 6.90 | 1.097 |
| Hay (all) | Pennsylvania | 320 | 9285 | 566802 | 6.05 | 5.11 | 1.184 |
| Hay (all) | South Carolina | 8 | 51 | 109312 | 4.61 | 5.15 | 0.896 |
| Hay (all) | South Dakota | 43 | 5064 | 1315789 | 4.50 | 4.59 | 0.980 |
| Hay (all) | Tennessee | 9 | 222 | 714980 | 5.42 | 4.93 | 1.100 |
| Hay (all) | Texas | 27 | 3319 | 2202429 | 9.47 | 4.84 | 1.958 |
| Hay (all) | Utah | 12 | 921 | 275304 | 6.41 | 7.88 | 0.812 |
| Hay (all) | Vermont | 175 | 8108 | 74899 | 5.11 | 3.76 | 1.357 |
| Hay (all) | Virginia | 35 | 2103 | 475709 | 7.50 | 5.11 | 1.469 |
| Hay (all) | Washington | 84 | 2610 | 352227 | 7.68 | 8.33 | 0.922 |
| Hay (all) | Wisconsin | 609 | 17944 | 663968 | 7.64 | 6.65 | 1.148 |
| Hay (all) | Wyoming | 14 | 2009 | 429150 | 7.21 | 4.75 | 1.519 |
| Haylage | California | 50 | 5257 | 631579 | 22.75 | 12.74 | 1.786 |
| Haylage | Idaho | 4 | 244 | 591093 | 15.30 | 8.47 | 1.807 |
| Haylage | Illinois | 7 | 188 | 218623 | 13.24 | 7.93 | 1.669 |
| Haylage | Iowa | 61 | 1166 | 493927 | 11.56 | 7.59 | 1.522 |
| Haylage | Michigan | 18 | 332 | 489879 | 14.15 | 6.88 | 2.059 |
| Haylage | Minnesota | 67 | 2660 | 894737 | 9.65 | 5.73 | 1.684 |
| Haylage | Missouri | 6 | 205 | 1445344 | 10.03 | 4.59 | 2.185 |
| Haylage | New York | 193 | 8299 | 729960 | 8.49 | 5.87 | 1.447 |
| Haylage | Ohio | 89 | 1847 | 417004 | 12.70 | 6.63 | 1.916 |
| Haylage | Pennsylvania | 150 | 4007 | 696356 | 9.20 | 5.93 | 1.551 |
| Haylage | South Dakota | 6 | 626 | 1327935 | 12.52 | 4.68 | 2.675 |
| Haylage | Texas | 6 | 413 | 2244939 | 18.19 | 4.93 | 3.691 |
| Haylage | Vermont | 95 | 6174 | 117409 | 11.27 | 5.80 | 1.942 |
| Haylage | Washington | 45 | 2251 | 376518 | 16.71 | 8.69 | 1.923 |
| Haylage | Wisconsin | 232 | 8950 | 1093117 | 12.05 | 7.93 | 1.520 |
| Hay (other) | Arkansas | 3 | 33 | 493927 | 5.26 | 4.48 | 1.175 |
| Hay (other) | California | 84 | 4594 | 202429 | 5.49 | 7.61 | 0.721 |
| Hay (other) | Colorado | 20 | 2070 | 242915 | 3.27 | 3.92 | 0.834 |
| Hay (other) | Georgia | 4 | 20 | 234818 | 2.69 | 5.82 | 0.462 |
| Hay (other) | Idaho | 14 | 575 | 121457 | 4.23 | 4.70 | 0.900 |
| Hay (other) | Illinois | 18 | 289 | 101215 | 6.65 | 6.05 | 1.100 |
| Hay (other) | Indiana | 40 | 568 | 145749 | 6.34 | 6.16 | 1.029 |
| Hay (other) | Kentucky | 22 | 392 | 850202 | 5.42 | 4.48 | 1.210 |
| Hay (other) | Maryland | 15 | 478 | 64777 | 4.10 | 5.38 | 0.762 |
| Hay (other) | Massachusetts | 40 | 468 | 26316 | 5.73 | 3.83 | 1.497 |
| Hay (other) | Michigan | 53 | 1261 | 137652 | 4.77 | 4.70 | 1.014 |
| Hay (other) | Minnesota | 106 | 2920 | 327935 | 5.80 | 3.58 | 1.619 |
| Hay (other) | Missouri | 36 | 1055 | 1295547 | 5.26 | 4.48 | 1.175 |
| Hay (other) | Montana | 23 | 1645 | 356275 | 2.89 | 3.81 | 0.759 |
| Hay (other) | Nebraska | 10 | 206 | 708502 | 7.61 | 3.36 | 2.267 |
| Hay (other) | New Jersey | 17 | 155 | 37247 | 2.42 | 5.15 | 0.470 |
| Hay (other) | New York | 272 | 12163 | 437247 | 6.00 | 4.03 | 1.489 |
| Hay (other) | North Dakota | 15 | 889 | 425101 | 6.74 | 4.26 | 1.584 |
| Hay (other) | Ohio | 140 | 2432 | 263158 | 5.82 | 5.60 | 1.040 |
| Hay (other) | Oregon | 54 | 4747 | 275304 | 4.93 | 5.38 | 0.917 |
| Hay (other) | Pennsylvania | 180 | 5957 | 425101 | 5.62 | 4.70 | 1.195 |
| Hay (other) | South Carolina | 8 | 51 | 109312 | 4.61 | 5.15 | 0.896 |
| Hay (other) | South Dakota | 17 | 934 | 546559 | 3.31 | 3.81 | 0.871 |
| Hay (other) | Tennessee | 9 | 222 | 708502 | 5.42 | 4.93 | 1.100 |
| Hay (other) | Texas | 18 | 1550 | 2145749 | 4.88 | 4.70 | 1.038 |
| Hay (other) | Utah | 4 | 98 | 64777 | 3.09 | 5.15 | 0.600 |
| Hay (other) | Vermont | 158 | 7270 | 60729 | 5.11 | 3.81 | 1.341 |
| Hay (other) | Virginia | 32 | 1981 | 445344 | 7.55 | 4.93 | 1.532 |
| Hay (other) | Washington | 58 | 1503 | 182186 | 4.57 | 6.27 | 0.729 |
| Hay (other) | Wisconsin | 226 | 6054 | 157895 | 6.54 | 4.26 | 1.537 |
| Hay (other) | Wyoming | 11 | 702 | 230769 | 3.52 | 3.81 | 0.924 |
| Hazelnut | Oregon | 7 | 74 | 12146 | 1.63 | 2.69 | 0.608 |
| Hops | Washington | 5 | 93 | 11683 | 1.29 | 2.17 | 0.596 |
| Lemon | California | 195 | 857 | 18623 | 28.47 | 36.62 | 0.777 |
| Lentil | Montana | 10 | 325 | 48178 | 0.88 | 1.66 | 0.532 |
| Lettuce (all) | Arizona | 10 | 1310 | 26113 | 19.70 | 36.62 | 0.538 |
| Lettuce (all) | California | 164 | 11333 | 79555 | 9.75 | 34.77 | 0.280 |
| Maize (grain) | California | 13 | 240 | 38462 | 10.23 | 10.35 | 0.989 |
| Maize (grain) | Colorado | 11 | 215 | 408907 | 7.49 | 9.16 | 0.818 |
| Maize (grain) | Georgia | 7 | 18 | 125506 | 5.42 | 10.66 | 0.509 |
| Maize (grain) | Idaho | 11 | 79 | 32389 | 10.24 | 12.54 | 0.816 |
| Maize (grain) | Illinois | 127 | 4195 | 4757085 | 8.75 | 12.54 | 0.697 |
| Maize (grain) | Indiana | 152 | 1476 | 2336032 | 7.06 | 11.79 | 0.599 |
| Maize (grain) | Iowa | 385 | 10037 | 5384615 | 7.20 | 11.16 | 0.645 |
| Maize (grain) | Kansas | 21 | 1490 | 1538462 | 9.47 | 9.34 | 1.013 |
| Maize (grain) | Kentucky | 15 | 85 | 578947 | 5.64 | 9.91 | 0.569 |
| Maize (grain) | Maryland | 22 | 1073 | 174089 | 7.73 | 10.97 | 0.704 |
| Maize (grain) | Michigan | 132 | 5290 | 894737 | 6.89 | 10.10 | 0.683 |
| Maize (grain) | Minnesota | 278 | 8839 | 3056680 | 6.48 | 9.78 | 0.662 |
| Maize (grain) | Missouri | 74 | 1511 | 1368421 | 6.20 | 11.66 | 0.532 |
| Maize (grain) | Montana | 3 | 1 | 30364 | 6.61 | 6.27 | 1.053 |
| Maize (grain) | Nebraska | 82 | 3762 | 3623482 | 9.21 | 11.23 | 0.820 |
| Maize (grain) | North Carolina | 10 | 302 | 315789 | 6.28 | 8.28 | 0.758 |
| Maize (grain) | North Dakota | 17 | 2113 | 1024291 | 4.42 | 7.78 | 0.568 |
| Maize (grain) | Ohio | 244 | 4106 | 1404858 | 5.97 | 11.04 | 0.541 |
| Maize (grain) | Oregon | 12 | 764 | 15789 | 6.41 | 11.91 | 0.538 |
| Maize (grain) | Pennsylvania | 256 | 4434 | 417004 | 7.22 | 9.66 | 0.747 |
| Maize (grain) | South Dakota | 27 | 1342 | 2153846 | 5.52 | 9.28 | 0.595 |
| Maize (grain) | Tennessee | 11 | 132 | 340081 | 5.79 | 10.54 | 0.550 |
| Maize (grain) | Texas | 15 | 1693 | 805668 | 6.89 | 9.28 | 0.742 |
| Maize (grain) | Washington | 9 | 774 | 44534 | 5.49 | 13.48 | 0.407 |
| Maize (grain) | Wisconsin | 539 | 8667 | 1259109 | 6.24 | 9.78 | 0.638 |
| Maize (grain) | Wyoming | 5 | 279 | 24291 | 9.13 | 8.65 | 1.055 |
| Maize (silage) | California | 15 | 1478 | 170040 | 50.50 | 58.23 | 0.867 |
| Maize (silage) | Colorado | 3 | 350 | 44534 | 48.82 | 55.99 | 0.872 |
| Maize (silage) | Idaho | 4 | 591 | 95142 | 47.32 | 62.71 | 0.755 |
| Maize (silage) | Illinois | 5 | 18 | 32389 | 41.59 | 49.27 | 0.844 |
| Maize (silage) | Indiana | 88 | 449 | 40486 | 41.03 | 49.27 | 0.833 |
| Maize (silage) | Iowa | 52 | 268 | 125506 | 37.63 | 44.79 | 0.840 |
| Maize (silage) | Kentucky | 10 | 62 | 30364 | 33.89 | 47.03 | 0.720 |
| Maize (silage) | Maine | 7 | 117 | 10931 | 31.38 | 41.48 | 0.756 |
| Maize (silage) | Michigan | 26 | 197 | 129555 | 27.23 | 45.91 | 0.593 |
| Maize (silage) | Minnesota | 61 | 1167 | 202429 | 32.74 | 40.31 | 0.812 |
| Maize (silage) | Missouri | 13 | 111 | 32389 | 28.38 | 40.31 | 0.704 |
| Maize (silage) | Nebraska | 4 | 112 | 105263 | 35.88 | 47.03 | 0.763 |
| Maize (silage) | Nevada | 3 | 2 | 1215 | 26.88 | 44.79 | 0.600 |
| Maize (silage) | New York | 143 | 2129 | 182186 | 30.91 | 40.31 | 0.767 |
| Maize (silage) | North Carolina | 3 | 34 | 20243 | 29.92 | 42.55 | 0.703 |
| Maize (silage) | Ohio | 95 | 635 | 76923 | 35.48 | 45.91 | 0.773 |
| Maize (silage) | Oregon | 8 | 293 | 16194 | 38.23 | 55.99 | 0.683 |
| Maize (silage) | Pennsylvania | 152 | 1190 | 165992 | 38.23 | 44.79 | 0.854 |
| Maize (silage) | Texas | 5 | 1087 | 85020 | 31.62 | 49.27 | 0.642 |
| Maize (silage) | Vermont | 8 | 185 | 34413 | 38.19 | 40.31 | 0.947 |
| Maize (silage) | Virginia | 7 | 81 | 50607 | 34.38 | 43.67 | 0.787 |
| Maize (silage) | Washington | 10 | 228 | 42510 | 45.84 | 62.71 | 0.731 |
| Maize (silage) | Wisconsin | 224 | 3167 | 344130 | 30.12 | 41.43 | 0.727 |
| Maize (sweet) | California | 42 | 128 | 12915 | 16.04 | 19.04 | 0.843 |
| Maize (sweet) | Florida | 3 | 3 | 13765 | 1.12 | 15.12 | 0.074 |
| Maize (sweet) | Illinois | 8 | 3 | 2470 | 5.81 | 14.12 | 0.412 |
| Maize (sweet) | Indiana | 5 | 3 | 2146 | 1.14 | 9.30 | 0.122 |
| Maize (sweet) | Maine | 9 | 2 | 648 | 7.52 | 7.84 | 0.960 |
| Maize (sweet) | Maryland | 4 | 1 | 1579 | 3.36 | 6.60 | 0.509 |
| Maize (sweet) | Michigan | 13 | 4 | 3725 | 9.19 | 10.08 | 0.912 |
| Maize (sweet) | New Jersey | 4 | 1 | 2429 | 7.88 | 10.08 | 0.781 |
| Maize (sweet) | New York | 24 | 42 | 7328 | 3.11 | 10.98 | 0.283 |
| Maize (sweet) | North Carolina | 11 | 11 | 1862 | 5.60 | 7.50 | 0.747 |
| Maize (sweet) | Ohio | 13 | 4 | 6113 | 3.95 | 9.74 | 0.405 |
| Maize (sweet) | Oregon | 39 | 1832 | 1984 | 16.18 | 16.45 | 0.983 |
| Maize (sweet) | Pennsylvania | 21 | 24 | 4575 | 7.13 | 7.84 | 0.909 |
| Maize (sweet) | Vermont | 27 | 40 | 368 | 9.25 | 5.66 | 1.635 |
| Maize (sweet) | Washington | 37 | 2168 | 4656 | 18.55 | 17.69 | 1.048 |
| Maize (sweet) | Wisconsin | 55 | 365 | 1417 | 9.80 | 12.54 | 0.781 |
| Oat | California | 17 | 642 | 4049 | 3.85 | 3.58 | 1.073 |
| Oat | Illinois | 46 | 1216 | 10121 | 2.86 | 2.87 | 0.999 |
| Oat | Indiana | 12 | 95 | 4049 | 1.48 | 2.65 | 0.559 |
| Oat | Iowa | 162 | 2603 | 22267 | 2.11 | 2.29 | 0.919 |
| Oat | Kansas | 4 | 33 | 6073 | 2.94 | 2.01 | 1.466 |
| Oat | Maine | 14 | 57 | 12551 | 1.58 | 2.51 | 0.631 |
| Oat | Michigan | 34 | 287 | 16194 | 1.61 | 2.47 | 0.652 |
| Oat | Minnesota | 103 | 2111 | 50607 | 1.76 | 2.26 | 0.780 |
| Oat | Missouri | 13 | 233 | 5263 | 2.55 | 2.33 | 1.097 |
| Oat | Montana | 6 | 965 | 6478 | 1.95 | 2.47 | 0.789 |
| Oat | Nebraska | 27 | 584 | 8097 | 1.83 | 2.87 | 0.640 |
| Oat | New York | 108 | 1194 | 16194 | 1.61 | 2.26 | 0.715 |
| Oat | North Carolina | 3 | 18 | 6883 | 1.14 | 2.40 | 0.475 |
| Oat | North Dakota | 31 | 2354 | 42510 | 2.14 | 2.62 | 0.820 |
| Oat | Ohio | 88 | 790 | 15789 | 1.57 | 2.26 | 0.695 |
| Oat | Oregon | 10 | 174 | 7287 | 1.83 | 3.05 | 0.600 |
| Oat | Pennsylvania | 56 | 396 | 24291 | 1.81 | 2.08 | 0.872 |
| Oat | South Dakota | 25 | 1079 | 40486 | 2.34 | 3.33 | 0.703 |
| Oat | Washington | 12 | 39 | 2024 | 1.28 | 2.51 | 0.508 |
| Oat | Wisconsin | 198 | 2054 | 56680 | 1.85 | 2.22 | 0.833 |
| Oat | Wyoming | 5 | 224 | 2834 | 2.07 | 2.11 | 0.977 |
| Onion | California | 43 | 374 | 17773 | 50.95 | 58.30 | 0.874 |
| Onion | Colorado | 8 | 1 | 1457 | 13.33 | 55.99 | 0.238 |
| Onion | Georgia | 6 | 3 | 4453 | 4.87 | 26.32 | 0.185 |
| Onion | Michigan | 8 | 1 | 1012 | 21.39 | 41.43 | 0.516 |
| Onion | New York | 25 | 12 | 3239 | 4.76 | 33.03 | 0.144 |
| Onion | Oregon | 19 | 4 | 7935 | 24.75 | 81.35 | 0.304 |
| Onion | Wisconsin | 58 | 26 | 688 | 21.76 | 52.10 | 0.418 |
| Orange | California | 147 | 1301 | 52632 | 13.98 | 26.67 | 0.524 |
| Orange | California | 151 | 676 | 14575 | 23.38 | 26.63 | 0.878 |
| Pea | Oregon | 24 | 1334 | 7935 | 7.21 | 4.73 | 1.526 |
| Peach | Arkansas | 3 | 1 | 263 | 13.44 | 2.24 | 6.000 |
| Peach | California | 142 | 668 | 17814 | 17.20 | 31.56 | 0.545 |
| Peach | Georgia | 4 | 0 | 4413 | 8.96 | 7.30 | 1.227 |
| Peach | Pennsylvania | 9 | 6 | 1619 | 0.81 | 8.38 | 0.096 |
| Peach | Texas | 7 | 1 | 1255 | 3.36 | 2.75 | 1.220 |
| Peach | Utah | 4 | 0 | 526 | 2.24 | 11.20 | 0.200 |
| Peach | Washington | 36 | 109 | 972 | 23.00 | 11.67 | 1.971 |
| Pear | California | 87 | 269 | 4494 | 14.13 | 38.14 | 0.371 |
| Pear | Michigan | 4 | 0 | 283 | 2.24 | 8.71 | 0.257 |
| Pear | New York | 4 | 2 | 405 | 0.56 | 11.98 | 0.047 |
| Pear | Oregon | 33 | 106 | 5830 | 17.76 | 33.59 | 0.529 |
| Pear | Washington | 118 | 706 | 8623 | 34.92 | 43.74 | 0.798 |
| Pepper, bell | California | 142 | 206 | 8866 | 30.40 | 47.26 | 0.643 |
| Pepper, bell | Florida | 11 | 56 | 4818 | 31.64 | 29.11 | 1.087 |
| Pepper, bell | Georgia | 16 | 1 | 1538 | 2.58 | 23.52 | 0.110 |
| Pepper, bell | Michigan | 18 | 2 | 567 | 4.65 | 29.11 | 0.160 |
| Pepper, bell | New Jersey | 23 | 13 | 1134 | 28.19 | 38.07 | 0.740 |
| Pepper, bell | North Carolina | 37 | 18 | 850 | 4.05 | 23.52 | 0.172 |
| Pepper, bell | Ohio | 52 | 13 | 1053 | 13.36 | 19.60 | 0.682 |
| Plum/prune | California | 128 | 939 | 7287 | 9.25 | 14.06 | 0.658 |
| Plum/prune | Oregon | 31 | 35 | 526 | 2.35 | 13.44 | 0.175 |
| Potato | California | 82 | 1161 | 13401 | 28.58 | 52.63 | 0.543 |
| Potato | Colorado | 15 | 1426 | 24211 | 33.52 | 43.44 | 0.772 |
| Potato | Florida | 5 | 2 | 11862 | 1.57 | 26.88 | 0.058 |
| Potato | Idaho | 13 | 31 | 129555 | 26.06 | 46.50 | 0.561 |
| Potato | Illinois | 19 | 3 | 2591 | 8.01 | 46.47 | 0.172 |
| Potato | Kansas | 11 | 2 | 1660 | 5.98 | 38.07 | 0.157 |
| Potato | Maine | 68 | 166 | 20445 | 20.03 | 32.47 | 0.617 |
| Potato | Maryland | 14 | 4 | 931 | 5.38 | 42.55 | 0.126 |
| Potato | Massachusetts | 31 | 9 | 1457 | 10.29 | 31.91 | 0.322 |
| Potato | Michigan | 17 | 2 | 17206 | 10.50 | 41.43 | 0.254 |
| Potato | Minnesota | 33 | 4 | 16599 | 12.93 | 44.79 | 0.289 |
| Potato | New York | 58 | 27 | 6397 | 5.61 | 30.79 | 0.182 |
| Potato | North Carolina | 36 | 13 | 5466 | 2.82 | 23.52 | 0.120 |
| Potato | Ohio | 42 | 8 | 607 | 9.06 | 31.35 | 0.289 |
| Potato | Oregon | 44 | 1257 | 15749 | 31.06 | 64.95 | 0.478 |
| Potato | Pennsylvania | 44 | 29 | 2105 | 10.31 | 30.79 | 0.335 |
| Potato | Texas | 14 | 11 | 8340 | 17.80 | 37.51 | 0.475 |
| Potato | Virginia | 17 | 10 | 1822 | 2.93 | 27.99 | 0.105 |
| Potato | Washington | 60 | 528 | 66802 | 60.59 | 68.87 | 0.880 |
| Potato | Wisconsin | 69 | 93 | 25911 | 33.22 | 45.91 | 0.724 |
| Proso millet | Colorado | 8 | 1932 | 101215 | 1.76 | 2.04 | 0.861 |
| Proso millet | Nebraska | 21 | 2200 | 44939 | 1.25 | 1.82 | 0.689 |
| Raspberry | California | 57 | 134 | 3036 | 14.46 | 22.32 | 0.648 |
| Raspberry | Oregon | 24 | 26 | 972 | 3.26 | 4.04 | 0.808 |
| Raspberry | Washington | 52 | 26 | 3988 | 3.53 | 8.30 | 0.425 |
| Rice | California | 46 | 4536 | 174494 | 6.15 | 9.61 | 0.640 |
| Rice | Texas | 34 | 4227 | 59514 | 4.60 | 8.22 | 0.559 |
| Safflower | Utah | 4 | 662 | 7287 | 0.58 | 1.11 | 0.523 |
| Snap bean | California | 100 | 281 | 1700 | 12.70 | 12.32 | 1.031 |
| Snap bean | Florida | 6 | 17 | 10769 | 6.61 | 5.60 | 1.181 |
| Snap bean | Georgia | 27 | 31 | 3603 | 8.44 | 4.48 | 1.884 |
| Snap bean | Maryland | 17 | 6 | 405 | 2.00 | 6.49 | 0.308 |
| Snap bean | Michigan | 31 | 65 | 1053 | 1.94 | 7.84 | 0.248 |
| Snap bean | New York | 59 | 18 | 3968 | 4.34 | 7.05 | 0.615 |
| Snap bean | New York | 6 | 0 | 8267 | 11.20 | 8.96 | 1.250 |
| Snap bean | North Carolina | 38 | 16 | 1741 | 3.86 | 4.48 | 0.863 |
| Snap bean | Tennessee | 10 | 1 | 2186 | 1.98 | 6.39 | 0.310 |
| Snap bean | Virginia | 10 | 1 | 688 | 4.87 | 5.14 | 0.948 |
| Snap bean | Wisconsin | 14 | 396 | 25951 | 7.12 | 10.91 | 0.653 |
| Sorghum (grain) | Kansas | 10 | 377 | 1093117 | 4.35 | 4.64 | 0.937 |
| Sorghum (grain) | Missouri | 6 | 175 | 29555 | 4.10 | 6.33 | 0.647 |
| Sorghum (grain) | Texas | 10 | 1164 | 910931 | 3.30 | 3.83 | 0.864 |
| Sorghum (silage) | Illinois | 4 | 32 | 405 | 36.17 | 40.31 | 0.897 |
| Sorghum (silage) | Missouri | 3 | 23 | 4049 | 25.15 | 38.07 | 0.661 |
| Sorghum (silage) | New Mexico | 8 | 1180 | 13360 | 19.04 | 29.11 | 0.654 |
| Sorghum (silage) | Texas | 8 | 2623 | 40486 | 26.09 | 31.35 | 0.832 |
| Soybean | Illinois | 111 | 3856 | 3959514 | 2.82 | 3.76 | 0.750 |
| Soybean | Indiana | 34 | 502 | 2222672 | 2.66 | 3.76 | 0.707 |
| Soybean | Iowa | 299 | 6371 | 3975709 | 2.46 | 3.46 | 0.711 |
| Soybean | Kansas | 14 | 277 | 1603239 | 1.48 | 2.42 | 0.611 |
| Soybean | Kentucky | 8 | 112 | 708502 | 2.06 | 3.23 | 0.639 |
| Soybean | Maryland | 14 | 790 | 204453 | 2.24 | 3.09 | 0.724 |
| Soybean | Michigan | 85 | 3900 | 866397 | 2.01 | 2.89 | 0.697 |
| Soybean | Minnesota | 153 | 4829 | 2943320 | 1.70 | 2.82 | 0.601 |
| Soybean | Missouri | 97 | 3251 | 2267206 | 2.27 | 3.12 | 0.726 |
| Soybean | Nebraska | 55 | 2258 | 2165992 | 2.77 | 3.63 | 0.764 |
| Soybean | New York | 123 | 2860 | 132389 | 1.92 | 3.02 | 0.634 |
| Soybean | North Carolina | 21 | 774 | 700405 | 2.18 | 2.69 | 0.810 |
| Soybean | North Dakota | 13 | 814 | 2376518 | 1.29 | 2.32 | 0.557 |
| Soybean | Ohio | 122 | 2677 | 1959514 | 2.42 | 3.53 | 0.687 |
| Soybean | Pennsylvania | 93 | 2328 | 244939 | 2.35 | 3.29 | 0.714 |
| Soybean | South Dakota | 24 | 1130 | 2068826 | 1.91 | 3.02 | 0.632 |
| Soybean | Tennessee | 3 | 126 | 651822 | 1.73 | 3.09 | 0.561 |
| Soybean | Texas | 4 | 437 | 56680 | 2.15 | 2.59 | 0.830 |
| Soybean | Wisconsin | 145 | 2402 | 724696 | 1.77 | 2.96 | 0.597 |
| Spinach | Arizona | 5 | 335 | 3239 | 7.80 | 17.36 | 0.449 |
| Spinach | New Jersey | 5 | 2 | 526 | 6.94 | 19.04 | 0.365 |
| Spinach | Texas | 14 | 4 | 607 | 6.56 | 16.80 | 0.390 |
| Squash | California | 257 | 874 | 2470 | 16.94 | 19.04 | 0.890 |
| Squash | Florida | 15 | 45 | 2753 | 6.06 | 13.17 | 0.460 |
| Squash | Georgia | 31 | 15 | 1296 | 4.73 | 16.80 | 0.282 |
| Squash | Michigan | 52 | 61 | 2429 | 3.34 | 22.40 | 0.149 |
| Squash | New Jersey | 27 | 28 | 1093 | 20.01 | 14.68 | 1.363 |
| Squash | New York | 73 | 76 | 1741 | 18.61 | 18.00 | 1.034 |
| Squash | North Carolina | 57 | 31 | 1215 | 8.33 | 6.05 | 1.378 |
| Squash | Ohio | 54 | 50 | 648 | 10.54 | 21.28 | 0.495 |
| Squash | Oregon | 58 | 841 | 607 | 19.73 | 29.04 | 0.679 |
| Squash | Tennessee | 5 | 3 | 336 | 12.16 | 5.80 | 2.097 |
| Squash | Texas | 21 | 17 | 607 | 18.78 | 10.08 | 1.863 |
| Strawberry | California | 139 | 1025 | 15223 | 23.78 | 67.53 | 0.352 |
| Strawberry | New York | 33 | 16 | 567 | 4.78 | 3.60 | 1.328 |
| Strawberry | North Carolina | 18 | 9 | 648 | 12.50 | 14.56 | 0.859 |
| Strawberry | Oregon | 26 | 12 | 729 | 6.34 | 14.81 | 0.428 |
| Strawberry | Washington | 51 | 26 | 648 | 5.07 | 10.08 | 0.503 |
| Sunflower | Nebraska | 3 | 289 | 13563 | 1.27 | 1.51 | 0.844 |
| Sunflower | North Dakota | 8 | 554 | 263158 | 1.28 | 1.46 | 0.873 |
| Sunflower | South Dakota | 5 | 898 | 211336 | 1.34 | 1.88 | 0.714 |
| Sweet potato | Arkansas | 4 | 0 | 1579 | 13.89 | 22.40 | 0.620 |
| Sweet potato | California | 29 | 1493 | 7692 | 39.94 | 30.79 | 1.297 |
| Sweet potato | North Carolina | 38 | 755 | 29150 | 18.81 | 24.64 | 0.763 |
| Sweet potato | Texas | 6 | 0 | 364 | 8.62 | 17.42 | 0.495 |
| Tomato | Alabama | 15 | 1 | 486 | 24.64 | 39.19 | 0.629 |
| Tomato | Arkansas | 12 | 2 | 364 | 41.77 | 19.04 | 2.194 |
| Tomato | California | 339 | 512 | 13077 | 15.10 | 35.28 | 0.428 |
| Tomato | California | 27 | 1834 | 117004 | 70.39 | 108.57 | 0.648 |
| Tomato | Florida | 26 | 279 | 13360 | 4.43 | 31.35 | 0.141 |
| Tomato | Georgia | 63 | 59 | 931 | 13.98 | 27.99 | 0.499 |
| Tomato | Indiana | 14 | 2 | 332 | 19.09 | 20.76 | 0.920 |
| Tomato | Michigan | 47 | 8 | 769 | 8.90 | 27.46 | 0.324 |
| Tomato | New Jersey | 43 | 25 | 1174 | 17.27 | 24.09 | 0.717 |
| Tomato | New York | 92 | 45 | 1053 | 7.05 | 13.44 | 0.525 |
| Tomato | Ohio | 78 | 17 | 1457 | 16.26 | 29.67 | 0.548 |
| Tomato | Pennsylvania | 65 | 67 | 1012 | 22.85 | 18.59 | 1.229 |
| Tomato | Tennessee | 16 | 4 | 1579 | 2.28 | 38.07 | 0.060 |
| Tomato | Texas | 33 | 9 | 316 | 31.55 | 11.20 | 2.817 |
| Tomato | Virginia | 35 | 17 | 931 | 3.94 | 31.35 | 0.126 |
| Walnut | California | 195 | 2326 | 117409 | 2.08 | 3.00 | 0.694 |
| Watermelon | Arkansas | 6 | 1 | 648 | 14.22 | 22.40 | 0.635 |
| Watermelon | California | 61 | 117 | 3603 | 17.72 | 63.83 | 0.278 |
| Watermelon | Florida | 8 | 14 | 7976 | 19.94 | 27.44 | 0.727 |
| Watermelon | Georgia | 13 | 7 | 7692 | 16.91 | 30.23 | 0.559 |
| Watermelon | Indiana | 5 | 0 | 3077 | 25.20 | 43.67 | 0.577 |
| Watermelon | Maryland | 5 | 4 | 1336 | 20.17 | 36.95 | 0.546 |
| Watermelon | North Carolina | 16 | 3 | 2227 | 1.78 | 23.52 | 0.076 |
| Watermelon | Texas | 15 | 102 | 8097 | 19.66 | 29.11 | 0.675 |
| Watermelon | Virginia | 4 | 1 | 263 | 3.53 | 22.40 | 0.158 |
| Wheat (all) | California | 37 | 3249 | 82996 | 3.06 | 5.58 | 0.548 |
| Wheat (all) | Colorado | 37 | 8708 | 954656 | 2.12 | 2.56 | 0.828 |
| Wheat (all) | Georgia | 5 | 21 | 93117 | 2.32 | 3.29 | 0.706 |
| Wheat (all) | Idaho | 36 | 4157 | 484211 | 2.43 | 5.26 | 0.462 |
| Wheat (all) | Illinois | 42 | 962 | 271255 | 2.74 | 4.50 | 0.608 |
| Wheat (all) | Indiana | 10 | 78 | 135628 | 2.32 | 5.11 | 0.454 |
| Wheat (all) | Iowa | 46 | 891 | 6073 | 2.53 | 3.29 | 0.769 |
| Wheat (all) | Kansas | 45 | 4881 | 3562753 | 1.57 | 1.88 | 0.836 |
| Wheat (all) | Kentucky | 5 | 33 | 206478 | 2.72 | 4.77 | 0.571 |
| Wheat (all) | Michigan | 49 | 1945 | 196356 | 3.33 | 4.97 | 0.670 |
| Wheat (all) | Minnesota | 50 | 1978 | 490688 | 3.03 | 3.68 | 0.823 |
| Wheat (all) | Missouri | 40 | 1214 | 299595 | 2.22 | 3.90 | 0.569 |
| Wheat (all) | Montana | 71 | 25224 | 2287449 | 1.92 | 2.49 | 0.773 |
| Wheat (all) | Nebraska | 55 | 7549 | 587045 | 2.21 | 3.29 | 0.672 |
| Wheat (all) | New Mexico | 5 | 743 | 42510 | 1.78 | 1.88 | 0.944 |
| Wheat (all) | New York | 58 | 1136 | 38462 | 1.95 | 4.23 | 0.460 |
| Wheat (all) | North Carolina | 24 | 995 | 311741 | 2.70 | 3.90 | 0.693 |
| Wheat (all) | North Dakota | 54 | 7366 | 3032389 | 1.73 | 3.11 | 0.555 |
| Wheat (all) | Ohio | 58 | 1211 | 220648 | 3.21 | 4.97 | 0.645 |
| Wheat (all) | Oklahoma | 8 | 963 | 1133603 | 0.89 | 1.14 | 0.776 |
| Wheat (all) | Oregon | 21 | 1170 | 331174 | 3.63 | 3.65 | 0.993 |
| Wheat (all) | Pennsylvania | 40 | 1161 | 60729 | 2.91 | 4.37 | 0.667 |
| Wheat (all) | South Dakota | 34 | 3073 | 957085 | 1.94 | 3.73 | 0.521 |
| Wheat (all) | Texas | 19 | 977 | 910931 | 2.02 | 2.02 | 1.000 |
| Wheat (all) | Utah | 25 | 12831 | 47368 | 1.08 | 3.38 | 0.320 |
| Wheat (all) | Virginia | 17 | 717 | 105263 | 3.03 | 4.57 | 0.662 |
| Wheat (all) | Washington | 21 | 961 | 910931 | 4.39 | 3.24 | 1.357 |
| Wheat (all) | Wisconsin | 103 | 1135 | 101215 | 2.98 | 4.37 | 0.682 |
| Wheat (all) | Wyoming | 32 | 10953 | 50607 | 2.10 | 2.55 | 0.823 |
| Wheat (durum) | California | 8 | 1161 | 10121 | 2.91 | 7.05 | 0.413 |
| Wheat (durum) | Montana | 7 | 1447 | 174089 | 1.72 | 2.08 | 0.824 |
| Wheat (durum) | North Dakota | 6 | 964 | 321862 | 1.41 | 2.39 | 0.591 |
| Wheat (spring) | Colorado | 6 | 1763 | 2834 | 2.73 | 4.91 | 0.555 |
| Wheat (spring) | Idaho | 21 | 2251 | 222672 | 2.67 | 4.22 | 0.632 |
| Wheat (spring) | Minnesota | 40 | 1356 | 647773 | 2.82 | 2.73 | 1.035 |
| Wheat (spring) | Montana | 64 | 18269 | 1113360 | 1.90 | 2.55 | 0.745 |
| Wheat (spring) | North Dakota | 44 | 5553 | 2510121 | 1.76 | 3.16 | 0.557 |
| Wheat (spring) | South Dakota | 26 | 1292 | 554656 | 2.26 | 3.52 | 0.642 |
| Wheat (spring) | Washington | 10 | 200 | 246964 | 1.36 | 2.55 | 0.531 |
| Wheat (winter) | California | 21 | 1467 | 72874 | 3.01 | 5.38 | 0.560 |
| Wheat (winter) | Colorado | 31 | 6945 | 951417 | 1.97 | 2.55 | 0.770 |
| Wheat (winter) | Georgia | 5 | 21 | 93117 | 2.32 | 3.29 | 0.706 |
| Wheat (winter) | Idaho | 17 | 1906 | 295547 | 2.15 | 5.38 | 0.401 |
| Wheat (winter) | Illinois | 42 | 962 | 271255 | 2.74 | 4.50 | 0.608 |
| Wheat (winter) | Indiana | 10 | 78 | 135628 | 2.32 | 5.11 | 0.454 |
| Wheat (winter) | Iowa | 40 | 844 | 6073 | 2.55 | 3.29 | 0.774 |
| Wheat (winter) | Kansas | 42 | 4878 | 3562753 | 1.57 | 1.88 | 0.836 |
| Wheat (winter) | Kentucky | 5 | 33 | 206478 | 2.72 | 4.77 | 0.571 |
| Wheat (winter) | Minnesota | 12 | 622 | 12955 | 3.50 | 3.29 | 1.062 |
| Wheat (winter) | Missouri | 40 | 1214 | 299595 | 2.22 | 3.90 | 0.569 |
| Wheat (winter) | Montana | 29 | 5509 | 906883 | 2.06 | 2.75 | 0.749 |
| Wheat (winter) | New York | 53 | 989 | 38462 | 1.93 | 4.23 | 0.456 |
| Wheat (winter) | North Carolina | 24 | 995 | 311741 | 2.70 | 3.90 | 0.693 |
| Wheat (winter) | North Dakota | 7 | 849 | 224696 | 1.87 | 3.29 | 0.568 |
| Wheat (winter) | Ohio | 46 | 949 | 220648 | 3.26 | 4.97 | 0.655 |
| Wheat (winter) | Oregon | 13 | 587 | 299595 | 3.84 | 3.70 | 1.038 |
| Wheat (winter) | Pennsylvania | 36 | 1134 | 60729 | 2.92 | 4.37 | 0.668 |
| Wheat (winter) | South Dakota | 12 | 1781 | 437247 | 1.71 | 3.70 | 0.464 |
| Wheat (winter) | Texas | 19 | 977 | 910931 | 2.02 | 2.02 | 1.000 |
| Wheat (winter) | Utah | 25 | 12831 | 44130 | 1.08 | 3.36 | 0.322 |
| Wheat (winter) | Virginia | 17 | 717 | 105263 | 3.03 | 4.57 | 0.662 |
| Wheat (winter) | Washington | 14 | 761 | 663968 | 5.19 | 3.49 | 1.487 |
| Wheat (winter) | Wisconsin | 87 | 1004 | 101215 | 3.15 | 4.37 | 0.720 |
| Wheat (winter) | Wyoming | 32 | 10953 | 50607 | 2.10 | 2.55 | 0.823 |
